# Edible Biopolymer Coatings for Postharvest Preservation in Warm‐Chain Horticultural Supply Chain: Mechanisms, Materials, Design Strategies, and Future Directions

**DOI:** 10.1111/1541-4337.70675

**Published:** 2026-10-18

**Authors:** Olaoluwa Omoniyi Olarewaju, Olufunke Omowumi Fajinmi

**Affiliations:** ^1^ School of Agriculture and Science University of KwaZulu‐Natal Scottsville South Africa

**Keywords:** biopolymer barrier properties, composite coating formulations, controlled‐release systems, food security, postharvest loss reduction, warm‐chain postharvest management

## Abstract

Fresh fruit and vegetable postharvest losses reach 30%–50% between harvest and consumption, concentrated in low‐ and middle‐income countries where warm‐chain supply chains (20°C–35°C, 55%–85% relative humidity, intermittent handling) replace the refrigerated cold chains conventional technology assumes. Edible coatings from polysaccharides, proteins, and lipids offer a non‐refrigeration postharvest intervention, with efficacy confirmed under controlled laboratory conditions, but commercial adoption remains marginal. This review argues that the gap between laboratory performance and field outcomes is primarily a systems‐design failure, in which existing formulations optimized for stable, refrigerated conditions are not engineered to maintain function under warm‐chain variability. Biopolymer materials face a three‐axis trade‐off, unable to simultaneously satisfy moisture barrier performance, gas exchange selectivity, and thermal‐hygroscopic stability, and all three degrade predictably under warm‐chain conditions through mechanisms current formulation design does not account for. Bioactive release kinetics compound this failure by depleting protection before supply chain completion, while the absence of a commodity classification framework makes the research literature noncumulative. This review makes three contributions. It provides, to the authors' knowledge, among the first integrated analyses of biopolymer coating performance failure under warm‐chain conditions, distinguished by its deployment‐focused approach rather than a materials‐cataloging one. It formalizes this into a three‐axis framework showing that composite design is physically necessary for warm‐chain applications, and proposes a four‐pillar warm‐chain design framework comprising predictive formulation modeling, controlled‐release calibration, commodity‐specific coating architecture, and standardized warm‐chain validation protocols. A six‐phase implementation pipeline and a commodity‐specific application matrix operationalize this framework into a demonstrable pathway from formulation to field deployment.

## 1 Introduction

Postharvest losses represent one of the most persistent inefficiencies in global food systems, with an estimated 30%–50% of fresh fruits and vegetables lost between harvest and consumption (FAO 2019, 2023; Hodges et al. 2011). These losses compound into producer income loss, reduced household dietary diversity, and wasted land, water, and energy inputs embedded in discarded produce (Parfitt et al. 2010). Losses are neither evenly distributed nor random. They are structurally concentrated in low‐ and middle‐income countries, particularly across sub‐Saharan Africa, where infrastructure deficits, fragmented supply chains, and limited access to postharvest technology create conditions of chronic vulnerability (Affognon et al. 2015). A global research network analysis confirms that postharvest technology innovation remains heavily skewed toward temperate, high‐income contexts, leaving tropical and subtropical production systems chronically underserved (Karoney et al. 2024).

The deterioration of harvested horticultural commodities is governed by a cascade of interconnected physiological and biochemical processes, including aerobic respiration, transpiration, ethylene biosynthesis, enzymatic senescence, and microbial colonization (Kader 2002; Hoffmann et al. 2025). These processes are intrinsically temperature‐sensitive. Even modest increases above optimal holding temperatures accelerate respiration rates, hasten ripening, and shorten marketable life (Hoffmann et al. 2025). Water loss is equally consequential, with even a 5% reduction in fresh weight producing measurable losses in firmness, turgor, and consumer appeal (Gidado et al. 2024). Postharvest management has therefore historically been anchored in temperature control, with refrigerated cold chains widely adopted as the primary strategy for suppressing metabolic activity and extending shelf life (Kitinoja et al. 2011).

However, cold‐chain dependence presupposes infrastructure that remains unavailable or unreliable across large portions of the developing world. In many tropical and subtropical production regions, fresh produce moves through what can be characterized as warm‐chain environments—supply chains where ambient temperatures routinely range between 20°C and 35°C, relative humidity fluctuates substantially, and intermittent or absent refrigeration means that commodities experience repeated thermal stress between harvest and sale (Kitinoja 2013; Ncama et al. 2018). This 20°C–35°C, 55%–85% relative humidity range is not drawn from a single established standard but is synthesized from postharvest storage and distribution studies conducted under non‐refrigerated conditions in tropical and subtropical production regions, reflecting the ambient conditions most consistently reported across the warm‐chain postharvest literature reviewed in this study (Hodges et al. 2011; Shakir et al. 2022). Under these conditions, the rate of physiological deterioration is substantially accelerated, and susceptibility to fungal and bacterial spoilage is markedly elevated (Parven et al. 2020). The social and economic dimensions of this reality are acute. Postharvest losses in warm‐chain environments disproportionately affect smallholder farmers and low‐income consumers who have the least capacity to absorb them (James and Zikankuba 2017). Despite this, most of the postharvest research continues to be designed and evaluated under controlled laboratory or refrigerated conditions, producing outcomes with limited transferability to warm‐chain supply chains (Figure 1).

**FIGURE 1:**
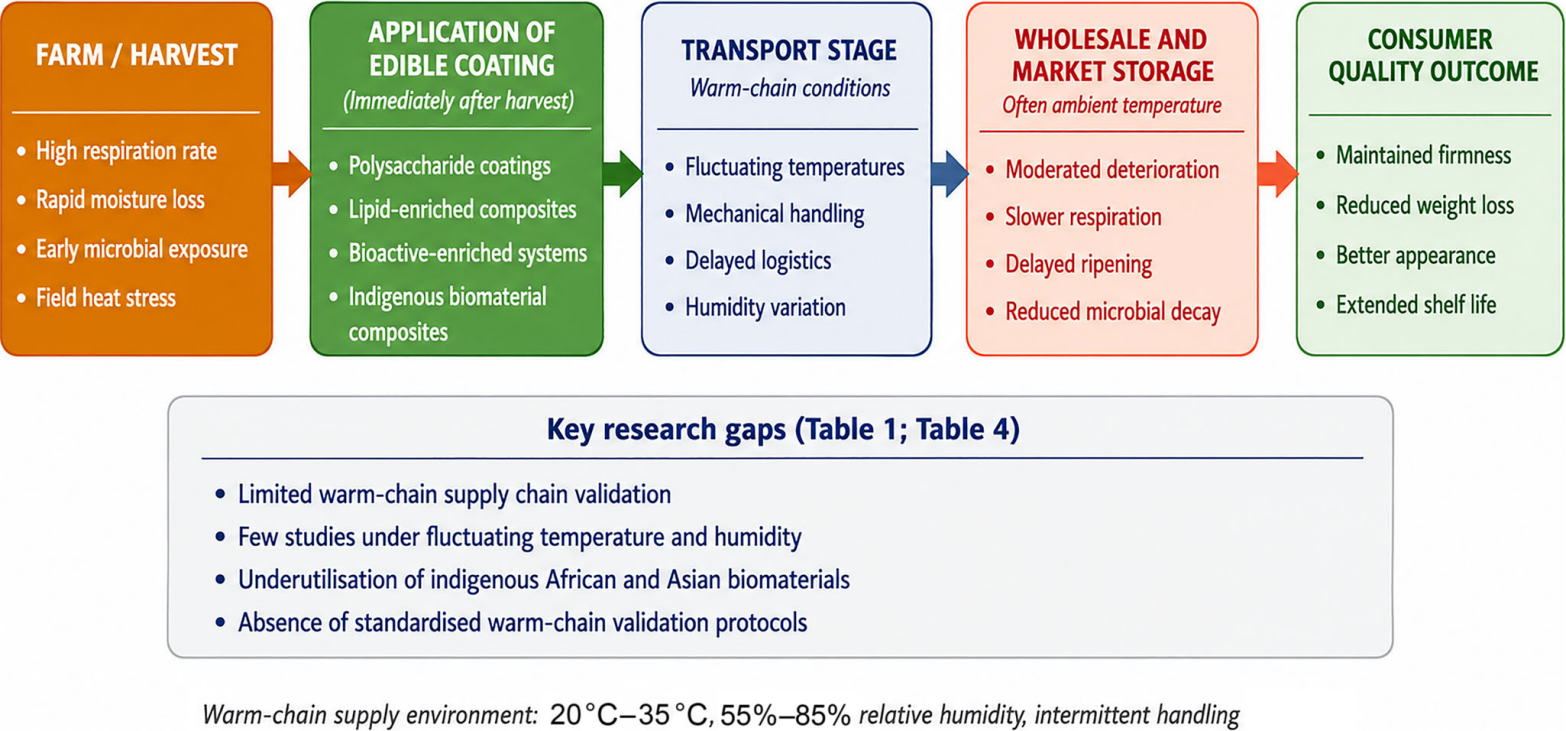
Role of edible biopolymer coatings across the warm‐chain horticultural supply chain, from harvest through coating application, ambient transport, and wholesale storage to the consumer, highlighting key research gaps identified in this review.

Edible coatings have drawn sustained research attention as a postharvest intervention that does not require refrigeration and can address multiple deterioration factors simultaneously. Primarily composed of polysaccharides, proteins, and lipids, these coatings create semipermeable barriers on the surface of commodities. This barrier regulates gas exchange, minimizes transpirational moisture loss, and inhibits microbial growth (M. Ali et al. 2025). Their versatility extends further, serving as carriers for antimicrobial and antioxidant compounds that provide active protection against spoilage organisms and oxidative degradation (Maringgal et al. 2020). The material diversity of edible coatings, such as chitosan, starch, alginate, gum arabic, wax composites, and protein films, offers a broad formulation space for commodity‐specific optimization (Kocira et al. 2021). Edible coatings are biodegradable, generally recognized as safe (GRAS), and cost‐compatible with low‐input production systems (Salehi 2020). Their technical accessibility without cold‐chain infrastructure makes them candidates for warm‐chain postharvest management, provided the formulation and validation challenges can be resolved. Edible coatings have been shown to delay ripening, reduce weight loss, lower respiration rates, and extend shelf life across tropical horticultural commodities under controlled storage conditions (Ncama et al. 2018).

However, the transition from laboratory promise to commercial reality has proven elusive. A central and underexamined reason for this gap is that most coating formulations are developed and validated under stable, controlled conditions that bear limited resemblance to the thermal and hygroscopic variability of real supply chains. Under fluctuating temperature and humidity, the structural and functional properties of biopolymer matrices diverge substantially from their laboratory‐characterized behavior. Gas permeability increases with rising temperature as polymer chain mobility increases, moisture barrier integrity is compromised under high humidity through swelling and plasticization, and bioactive compounds degrade or are released prematurely (Jafarzadeh et al. 2021). Studies evaluating coatings under ambient storage conditions consistently report diminished performance relative to refrigerated controls, confirming that temperature and humidity variability are not minor perturbations but primary determinants of coating functionality (Thakur et al. 2018). Variability in handling practices, packaging interfaces, and transportation conditions further compounds this instability, producing inconsistent outcomes even when the same formulation is applied to the same commodity (Moalemiyan and Ramaswamy 2012). The cumulative evidence points to a systems‐design failure rather than a materials discovery deficit. The problem is not that effective coating materials are unavailable, but that the design, optimization, and validation frameworks in common use are misaligned with the environments in which coatings are most urgently needed.

Five review papers define the current horizon of edible coating science and establish this review's position. Dhall (2013) provided the first comprehensive treatment of polysaccharide, protein, lipid, and composite coatings as functional ingredient carriers, but evaluated coating performance only under controlled storage conditions and did not address biopolymer barrier properties under thermal or hygroscopic stress. Otoni et al. (2017) organized the field around functional property relationships of edible films based on fruit and vegetable materials, establishing rigorous structure–property connections, but framed the field as a materials catalog rather than deriving what warm‐chain deployment conditions require. Arnon‐Rips and Poverenov (2018) proposed a layer‐by‐layer coating architecture framework that advanced composite design thinking, reasoning from physical architecture toward functional outcomes, but did not address warm‐chain supply chain requirements or integrate commodity physiological parameters. Ncama et al. (2018) reviewed plant‐based edible coatings in postharvest quality management for sub‐Saharan Africa. Still, they framed the problem as materials selection rather than systems design failure and did not examine why laboratory‐validated coatings underperform in warm‐chain conditions. M. Ali et al. (2025) provided the most recent synthesis of global edible coating advances, confirming materials progress, but organized the review around material categories rather than deployment conditions, leaving the warm‐chain performance gap undiagnosed. These reviews establish that the field has advanced in materials understanding while remaining anchored in a laboratory‐centered paradigm that treats warm‐chain conditions as validation context rather than primary design input. This review departs from that paradigm by proposing a four‐pillar warm‐chain design framework, grounded in polymer diffusion physics, controlled‐release kinetics, commodity physiology, and supply chain realities, that reverses reasoning from what materials can do to what warm‐chain environments require.

This review critically examines edible biopolymer coating technologies through the lens of warm‐chain applicability. It synthesizes current mechanistic understanding of how coating properties interact with dynamic environmental conditions, evaluates the structural and functional limitations of conventional biopolymer systems under nonideal storage, and analyzes advanced formulation strategies that have shown potential for improving real‐world performance. Building on this, the review identifies the most consequential research gaps and proposes a warm‐chain design framework organized around four priorities, comprising predictive formulation modeling, controlled‐release systems calibrated to postharvest timelines, commodity‐specific coating architectures, and standardized warm‐chain validation protocols. This review demonstrates that this gap is not principally a consequence of insufficient material diversity, but of design and validation strategies that remain detached from the coupled barrier, physiological, and environmental constraints of warm‐chain supply systems.

## 2 Literature Search and Selection

Six targeted searches of the Scopus database were conducted, each addressing a distinct thematic domain of the review. Search A covered edible coatings on fresh horticultural produce and postharvest preservation (2500 records). Search B targeted ambient and warm‐chain storage conditions (328 records). Search C targeted biopolymer film barrier and permeability properties (1443 records). Search D targeted indigenous biopolymer materials including gum arabic, aloe gel, *Opuntia* mucilage, and related materials (817 records). Search E targeted composite and active edible coating systems (108 records). Search F targeted edible coatings evaluated specifically at ambient temperatures including 25°C and 28°C (223 records). All searches were restricted to English‐language publications between 2010 and 2025 across relevant Scopus subject areas including agricultural science, biochemistry, chemistry, materials science, and environmental science.

The six searches returned 5419 records in total. After deduplication by title, 2840 unique records remained. Title and abstract screening using the following criteria reduced this to a final set of 150 studies for full synthesis, comprising 87 original research articles and 63 review articles. Inclusion criteria: (1) studies reporting edible coating or edible film application to fresh horticultural commodities, including intact fruit, vegetables, or fresh‐cut produce; (2) studies reporting at least one measurable postharvest quality, physiological, or barrier property outcome; (3) studies based on polysaccharide, protein, lipid, or composite biopolymer coating systems; (4) English‐language peer‐reviewed articles or review papers published between 2010 and 2025. Exclusion criteria: (1) studies reporting coating applications to processed, dried, fermented, or thermally treated products; (2) studies reporting coating material synthesis or film characterization without application to a horticultural commodity; (3) studies focused on grains, seeds, or nonfood crop products; (4) conference abstracts, theses, book chapters, and gray literature; (5) studies reporting exclusively on synthetic polymer coatings without a biopolymer component.

The 150 studies form the evidence base. Studies that contributed to screening and thematic classification without directly supporting a specific cited claim are not individually cited. Three foundational references published before 2010, Crank (1975), Saltveit (1999), and Talja et al. (2007), were retained to provide the polymer physics and postharvest physiology principles underpinning the review's analytical arguments. Narrative synthesis was selected over meta‐analysis because heterogeneity in outcome metrics, storage conditions, and commodity types across the 150 studies precluded meaningful quantitative pooling.

## 3 Mechanistic Basis of Edible Coatings Under Dynamic Conditions

These mechanisms explain why coatings designed under controlled laboratory conditions cannot perform reliably in warm‐chain environments. Coating effectiveness depends on the ability to regulate mass transfer and modulate physiological, biochemical, and microbial processes at the commodity–environment interface, including gas exchange, water vapor transfer, respiration and ethylene metabolism, and microbial and oxidative processes. These functions are well studied under controlled conditions, but their behavior under fluctuating temperature and humidity is less predictable (Figure 2). Figure 2 maps six deterioration pathways that coating systems must address, showing how warm‐chain conditions amplify each pathway in compounding ways.

**FIGURE 2:**
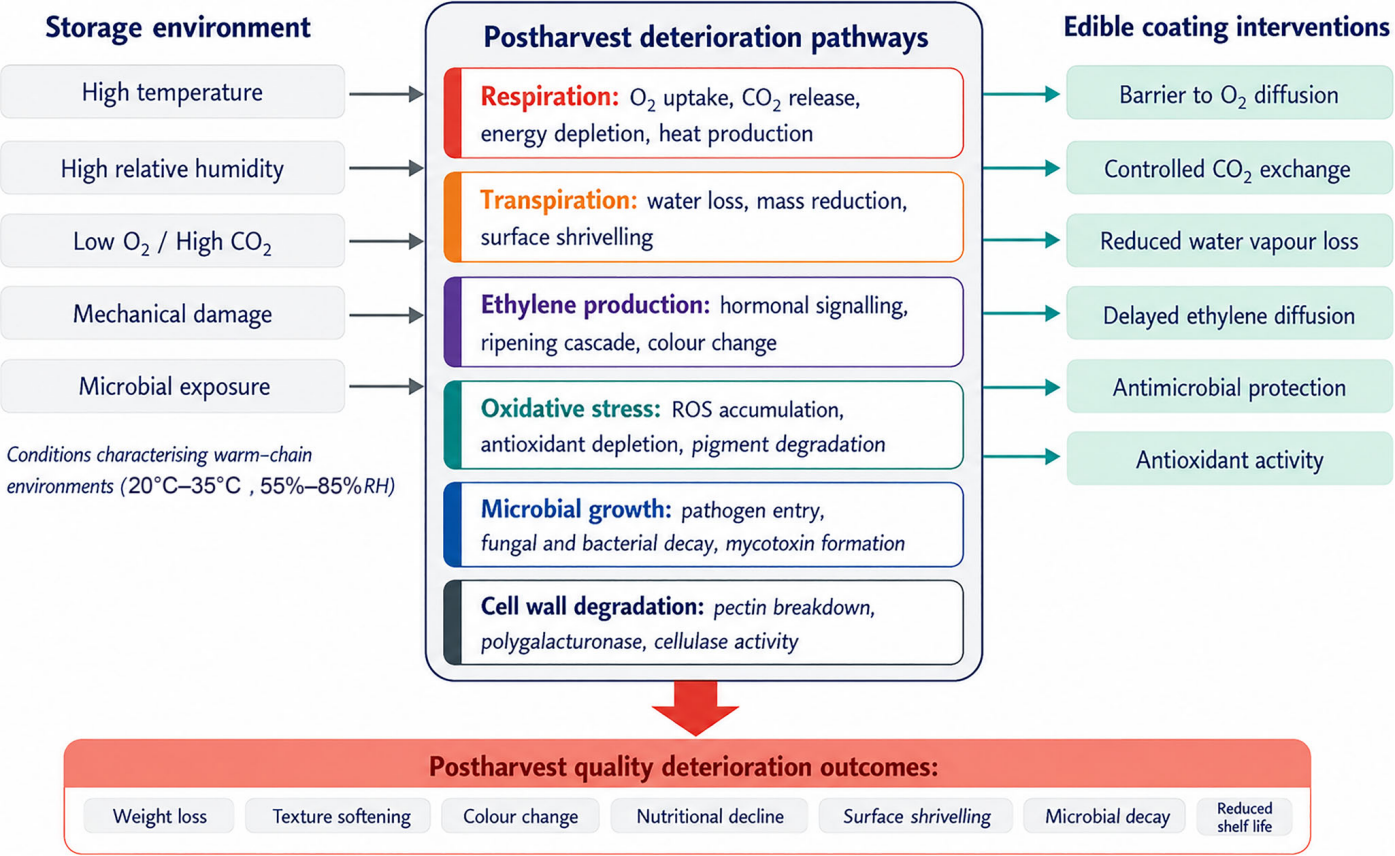
Physiological and biochemical deterioration pathways activated under warm‐chain storage conditions (20°C–35°C, 55%–85% RH) and the corresponding coating‐based interventions.

### 3.1 Gas Exchange and Internal Atmosphere Regulation

Edible coatings act as semipermeable barriers that regulate the diffusion of oxygen (O_2_) and carbon dioxide (CO_2_), thereby modifying the internal atmosphere of fresh produce and influencing respiration and ripening processes. Coating permeability depends strongly on polymer composition, molecular structure, and interactions within the film matrix. Polysaccharide‐based coatings show low oxygen permeability under dry conditions due to dense hydrogen‐bonded networks. Still, this barrier property degrades substantially as humidity increases toward warm‐chain levels. In contrast, protein‐based coatings offer moderate permeability based on structure, and lipid components provide a hydrophobic barrier that trades gas permeability for moisture control (Table 1) (Kocira et al. 2021; Panahirad et al. 2021). Table 1 maps this trade‐off across material classes and shows that no class excels on all three performance axes, a pattern that the composite coating literature has attempted to resolve. Lipid‐based coatings, although inherently more permeable to gases, can be incorporated into composite systems to improve barrier selectivity and overall performance (Dhall 2013; Sapper and Chiralt 2018).

**TABLE 1:** Biopolymer barrier properties, thermal and hygroscopic behavior, and warm‐chain design implications.

Biopolymer or system	Chemical class	WVP range (g mm/m^2^ day kPa)	O_2_ barrier performance	Response to temperature increase (20°C–35°C)	Response to high humidity (above 70% RH)	Warm‐chain suitability	Warm‐chain design implication	References
Starch (native, unmodified)	Neutral polysaccharide	High (2.1–6.8)	Low to moderate; collapses above 75% RH	WVP increases sharply; chain mobility accelerates both gas and moisture diffusion	Severe swelling and structural softening; barrier dissolution above 80% RH	Poor	Not suitable as standalone warm‐chain coating; cross‐linked or hydrophobically modified variants required; composite with CMC, lipid, or cross‐linking agent essential before warm‐chain application	Abdillah and Charles (2021); Sapper and Chiralt (2018); Thakur et al. (2018)
Alginate (uncross‐linked)	Anionic polysaccharide	High (3.0–7.5)	Low when dry; collapses rapidly under humid conditions	WVP increases; film softens and loses mechanical integrity above 30°C without ionic cross‐linking	Rapid gel formation and swelling; film dissolution under repeated wet‐dry cycling; structural irreversibility under thermal cycling	Poor without cross‐linking	Divalent cation cross‐linking (Ca^2+^) is non‐negotiable for warm‐chain application; cross‐linked systems show substantially improved thermal and hygroscopic stability and represent the design baseline for alginate warm‐chain use	Otoni et al. (2014); Pirozzi et al. (2020); Y. Zhang et al. (2024)
Protein films (gelatin, whey protein)	Animal‐derived protein	Moderate to high (2.0–5.0)	Moderate at low RH; severe decline under humid conditions	WVP increases substantially; gelatin softens and deforms above 30°C–35°C; barrier function effectively lost at upper warm‐chain range	Structural denaturation and film dissolution above 80% RH; irreversible structural changes under repeated thermal cycling	Poor	Gelatin not suitable as primary warm‐chain barrier; application limited to composite systems contributing mechanical strength at lower temperatures; plant proteins (zein, soy isolate) with greater thermal stability warrant systematic warm‐chain investigation	Galus et al. (2020); Moosavi‐Nasab et al. (2023); Radi et al. (2017)
Chitosan	Cationic polysaccharide	Moderate to high (1.5–4.2)	Low to moderate; effective at low RH; barrier collapses above 65% RH	WVP increases progressively above 20°C; antimicrobial activity partially preserved through cationic membrane‐disruption mechanism	Severe plasticization; loss of barrier properties at tropical humidity levels; matrix swelling documented across multiple commodity studies	Moderate (antimicrobial more thermally stable than barrier)	Cannot function reliably as warm‐chain moisture barrier as a standalone material; composite with lipid component or ionic cross‐linking required; intrinsic cationic antimicrobial activity is retained under warm conditions and represents the primary warm‐chain functional contribution	Kerch (2015); Nair et al. (2020); Saberi Riseh et al. (2023)
Cellulose derivatives (CMC, HPC, HPMC)	Modified polysaccharide	Moderate (1.8–3.5)	Low to moderate; more stable across humidity range than starch or chitosan	WVP increases moderately; structural stability superior to starch and chitosan across the warm‐chain range	Moderate swelling; microcrack formation under RH cycling; structural integrity maintained to 75% RH under warm conditions	Moderate	Most thermally stable of the polysaccharide class; preferred base polymer for warm‐chain polysaccharide‐dominant systems; enrichment with moringa leaf extract or plant‐derived bioactives improves functional performance without compromising structural stability	Deng et al. (2017); Tesfay et al. (2017); Yildirim‐Yalcin et al. (2022)
Gum arabic (*Acacia* spp.)	Branched anionic polysaccharide‐protein complex	Moderate (1.2–2.8)	Moderate; branched architecture limits humidity‐induced structural collapse	WVP increases moderately; thermal stability superior to linear polysaccharides; film properties maintained at 25°C–30°C	Better humidity resistance than linear polysaccharides due to branched molecular architecture; integrity maintained to 75% RH	Good	Priority indigenous material for warm‐chain coating development; branched architecture confers intrinsic resilience to warm‐chain thermal and hygroscopic stresses; functional performance amplified by composite development with chitosan or plant‐derived bioactives	Daisy et al. (2020); Mahfoudhi and Hamdi (2015); Tiamiyu et al. (2023)
*Aloe vera* gel	Indigenous polysaccharide‐phenolic complex	Moderate (1.5–3.0)	Moderate; acemannan network provides functional O_2_ restriction; intrinsic bioactive compounds supplement passive barrier	Structural barrier broadly stable across warm‐chain range; phenolic bioactives degrade above 30°C, reducing antimicrobial and antioxidant functional advantage	Moderate swelling: compositional variability across gel sources creates inconsistent humidity response between batches	Good (barrier) Moderate (bioactive)	Standardized gel quality benchmarking and thermal stability characterization essential before warm‐chain deployment; composite with chitosan or gum arabic improves structural stability without compromising residual bioactive activity	Kahramanoğlu et al. (2019); Nicolau‐Lapeña et al. (2021); Sarker and Grift (2021)
*Opuntia* mucilage	Indigenous mucilaginous polysaccharide	Moderate to low (1.0–2.5)	Low to moderate; dense mucilage network limits gas diffusion; natural adhesive properties improve surface coverage	Largely uncharacterized above 25°C; single warm‐chain study confirms functionality at ambient temperature	Natural gel‐forming capacity may partially self‐seal micro‐discontinuities; full hygroscopic response profile uncharacterized	Promising but critically under‐characterized	Thermal and hygroscopic behavior across the full 20°C–35°C and 55%–85% RH warm‐chain range entirely absent from the literature; systematic physicochemical profiling under warm‐chain conditions is the immediate research priority before further application studies are conducted	Allegra et al. (2016); C. Rodrigues et al. (2023); Shinga and Fawole (2023)
Lipid and wax components (beeswax, carnauba wax, shellac)	Hydrophobic lipid and ester	Low (0.1–0.8)	High permeability; poor gas barrier; anaerobic risk when applied at thicknesses sufficient for moisture control	Crystalline structure thermally stable well below melting point (62°C–85°C); most thermally stable barrier class across the entire warm‐chain temperature range	Highly resistant to humidity‐induced plasticization; structural integrity maintained across the full warm‐chain humidity range	Excellent (moisture barrier only)	Must always be incorporated within composite systems, never as standalone coating; lipid fraction must be precisely calibrated so that the moisture barrier benefit is achieved without inducing anaerobic internal atmosphere, particularly critical at elevated warm‐chain respiration rates in climacteric fruits	Dhall (2013); Sapper and Chiralt (2018); Velickova et al. (2013)

*Note*: WVP values and material properties are compiled from the 150 reviewed studies; warm‐chain design implications are synthesized assessments derived by the authors from the reviewed evidence. Ordered from most to least thermally and hygroscopically vulnerable under warm‐chain conditions (20°C–35°C, 55%–85% RH). WVP = water vapor permeability reported as g mm/m^2^ day kPa. Warm‐chain suitability integrates thermal stability, humidity response, and available warm‐chain validation evidence. ↑ = increases; ↓ = decreases; ↔ = broadly stable. WVP values are compiled from reviewed studies reported under variable laboratory measurement conditions; direct cross‐material comparison requires matched measurement conditions, and the ranges should be interpreted as indicative rather than precisely equivalent.

Coatings reduce respiration rates by limiting oxygen availability and increasing tissue carbon dioxide concentration. For instance, chitosan‐ and alginate‐based coatings have been shown to delay ripening and maintain quality in fruits such as strawberries, plums, and papaya under refrigerated conditions (Gol et al. 2013; Velickova et al. 2013). Similar effects on ripening suppression and quality retention have been documented for mango and papaya coated with gum‐ and aloe‐based formulations under ambient storage conditions (Ebrahimi and Rastegar 2020; Parven et al. 2020). This confirms that gas regulation is achievable across material types and commodity categories.

However, gas permeability is highly sensitive to environmental conditions. Temperature increases molecular mobility within the coating polymer matrix, raising diffusion coefficients and progressively weakening the barrier effect as storage temperatures rise above those assumed during formulation (Flores‐López et al. 2016; Jafarzadeh et al. 2021). This temperature dependence follows an Arrhenius relationship, in which the diffusion coefficient increases exponentially with temperature according to $D=D_{0}\exp\left(-E_{a}/RT\right)$, where *E*_a_ is the activation energy for diffusion in each polymer–permeant system, making diffusion coefficients calculable across temperatures from experimental parameters (Crank 1975). Under elevated temperatures, increased respiration rates may exceed the regulatory capacity of the coating, resulting in internal atmosphere imbalances that accelerate deterioration rather than retard it. Conversely, excessively restrictive coatings can induce hypoxic conditions that trigger anaerobic respiration, leading to ethanol accumulation and off‐flavor development (Wilson et al. 2019; Yousuf et al. 2018). The temperature dependence of gas diffusivity across biopolymer coating matrices is therefore not a peripheral complication of warm‐chain storage. It is a fundamental design constraint, because a coating formulated to provide optimal gas regulation at 20°C will progressively over‐restrict oxygen as temperature rises and commodity respiration accelerates, creating the precise conditions for anaerobic deterioration that coating application was intended to prevent. Material improvement alone cannot resolve this. Permeability targets must instead be specified as functions of temperature rather than fixed parameters, connecting gas diffusivity directly to commodity respiration dynamics and the warm‐chain temperature range (Table 1).

### 3.2 Water Vapor Transfer and Moisture Dynamics

Moisture loss through transpiration is a major determinant of postharvest quality, affecting fresh weight, firmness, and visual appeal. Edible coatings reduce water vapor loss by forming diffusion barriers at the commodity surface, though effectiveness varies substantially with material composition and environmental conditions. Hydrophilic coatings based on polysaccharides and proteins exhibit high water vapor permeability due to their affinity for water molecules, which facilitates diffusion through the matrix and limits their effectiveness as moisture barriers (Kocira et al. 2021; N. Kumar and Neeraj 2019). In contrast, lipid‐based coatings including waxes and shellac composites provide superior moisture barriers by virtue of their hydrophobic nature, although their application may compromise gas exchange if not properly formulated (Tavassoli‐Kafrani et al. 2022). Composite coatings combining hydrophilic and hydrophobic components have therefore been developed to balance moisture retention and gas permeability, with multilayer and emulsion‐based systems offering improved performance relative to single‐component alternatives (Jafarzadeh et al. 2021; Otoni et al. 2017).

Coatings significantly reduce weight loss and maintain firmness across a range of commodities. Chitosan‐beeswax composite systems have demonstrated consistent reductions in water loss in strawberries, contributing to improved texture and extended marketable life (Gol et al. 2013; Velickova et al. 2013). Starch‐based systems have shown comparable effects in plums stored at warm ambient temperatures (Thakur et al. 2018). *Aloe vera* and gum arabic coatings have also been reported to reduce transpiration and preserve tissue hydration by forming cohesive film matrices over the commodity surface (Daisy et al. 2020; Kahramanoğlu et al. 2019). Under fluctuating humidity conditions, however, coating performance becomes less stable. High relative humidity induces swelling and plasticization of hydrophilic matrices, progressively increasing permeability and reducing structural coherence over time (Arnon‐Rips and Poverenov 2018; Otoni et al. 2017). Conversely, low relative humidity can accelerate dehydration of the coating film itself, promoting microcrack formation that creates pathways for direct moisture loss from the commodity surface (Armghan Khalid et al. 2022; Deng et al. 2017). The key feature of this moisture dynamic is its asymmetry. When elevated relative humidity demands effective moisture barrier performance from a coating, hydrophilic coating matrices absorb water, swell, and lose the structural coherence that enables barrier performance. This means that coatings tested under low‐humidity lab conditions overestimate their field moisture‐barrier effectiveness, increasingly so as ambient humidity rises.

The structural response of biopolymer coating matrices to temperature and humidity is governed by a material transition not systematically considered in current design. Each biopolymer matrix has a glass transition temperature (*T*_g_) below which it acts as a glassy solid with restricted mobility and stable barrier properties. Above *T*_g_, the matrix becomes rubbery, increasing chain mobility and diffusion while degrading barrier properties (Roos and Drusch 2015). Under dry conditions, biopolymer coating materials have *T*_g_ values at or above warm‐chain temperatures. Water acts as a plasticizer, depressing *T*_g_ relative to absorbed moisture content, such that at the 55%–85% RH characteristic of warm‐chain environments, the plasticized *T*_g_ of hydrophilic biopolymers including starch, chitosan, and protein‐based films shifts below the warm‐chain range (Talja et al. 2007). These materials therefore operate in the rubbery state during warm‐chain storage, since moisture plasticization depresses *T*_g_ below ambient temperature before thermal effects act, and the barrier performance losses across hydrophilic coating systems result from this transition. Defining the plasticized *T*_g_ of candidate biopolymer systems across warm‐chain humidity is a prerequisite for specifying temperature‐dependent permeability targets, forming the foundation for predictive formulation modeling in the warm‐chain design framework (Sapper and Chiralt 2018).

### 3.3 Respiration, Ethylene, and Metabolic Regulation

Respiration governs the rate of postharvest deterioration by determining substrate utilization and heat generation within plant tissues. By modifying internal gas composition, edible coatings indirectly suppress respiration rates and delay the onset of senescence. Reduced oxygen availability and elevated carbon dioxide concentrations within coated produce have been associated with lower metabolic activity and extended shelf life across diverse commodity types (Gol et al. 2013; Velickova et al. 2013).

Coatings can effectively slow metabolic processes in a range of fruits. Chitosan applications have been shown to significantly reduce carbon metabolism and delay softening in mango and banana under warm ambient conditions (Cosme Silva et al. 2017; Lustriane et al. 2018). Starch‐based and alginate coatings have similarly been effective in maintaining quality and reducing respiration rates in plums and fresh‐cut fruits stored under controlled conditions (Oms‐Oliu et al. 2008; Thakur et al. 2018). Daisy et al. (2020) and Shakir et al. (2022) documented that gum arabic‐based coatings delay ripening and reduce ethylene production in tomatoes, with concurrent maintenance of firmness and antioxidant capacity. Ethylene plays a critical role in ripening climacteric fruits, and its production and perception are influenced by coating‐induced changes in internal atmosphere (Kader 2002). Elevated CO_2_ within coated produce suppresses ethylene action through competitive inhibition at the ethylene receptor, reducing climacteric tissues' sensitivity to ethylene independently of production rate. In contrast, reduced O_2_ availability slows ethylene biosynthesis through inhibition of 1‐aminocyclopropane‐1‐carboxylic acid oxidase activity (Saltveit 1999). These mechanisms are distinct in their effects on ripening suppression, meaning coating design for climacteric warm‐chain commodities must account for both O_2_ and CO_2_ permeability rather than optimizing for gas restriction alone. Coatings that restrict gas exchange without maintaining CO_2_ balance for receptor‐level inhibition will produce weaker ripening suppression than formulations designed around the full modified atmosphere requirement. Composite and multilayer coatings have been explored as strategies to achieve more controlled modulation of ethylene‐related processes while avoiding the hypoxic risks associated with single‐layer restrictive formulations (Arnon‐Rips and Poverenov 2018; Otoni et al. 2017).

Under fluctuating temperature conditions, respiration rates vary dynamically, creating progressive changes in internal gas composition that a fixed‐permeability coating cannot accommodate. Elevated temperatures accelerate metabolic activity, while subsequent cooling may partially suppress respiration, resulting in cumulative stress effects that compound deterioration over time (Bhan et al. 2022; Chen et al. 2023). For climacteric fruits, this creates a compounding regulatory failure under warm‐chain temperature fluctuation. Rising temperature simultaneously accelerates ethylene‐driven ripening, increases coating gas permeability, and reduces the coating's capacity to maintain the modified internal atmosphere on which ripening suppression depends. The coating therefore loses its regulatory effectiveness at exactly the moment when the commodity's ethylene physiology most demands it, and recovers partially as temperature falls, only to lose it again in the next thermal cycle. A fixed‐permeability coating cannot accommodate this dynamic. Coating design for climacteric warm‐chain commodities requires that gas permeability specifications account explicitly for the commodity's ethylene production rate across the full temperature range of the supply chain, not merely at the reference temperature of the laboratory.

### 3.4 Microbial Growth and Oxidative Processes

Microbial spoilage is a major driver of postharvest losses, particularly under warm‐chain conditions where elevated temperature and humidity create environments highly conducive to fungal and bacterial proliferation. Edible coatings mitigate microbial growth through complementary physical and biochemical mechanisms. As physical barriers, coatings reduce the direct exposure of the commodity surface to environmental contaminants, while the incorporation of antimicrobial agents provides active protection against spoilage organisms (Romanazzi et al. 2017; Valencia‐Chamorro et al. 2011).

Biopolymer‐based coatings incorporating chitosan, essential oils, or plant‐derived polyphenols have demonstrated strong antimicrobial activity against a broad spectrum of postharvest pathogens (Nair et al. 2020; Pandey et al. 2022). Studies across diverse commodities including strawberries, papaya, and fresh‐cut fruits confirm that antimicrobial coatings can significantly reduce surface microbial loads and delay the onset of visible decay (Oms‐Oliu et al. 2008; Parven et al. 2020; Sogvar et al. 2016). *Aloe vera*‐based coatings have been reported to suppress microbial proliferation while simultaneously maintaining physicochemical quality attributes, reflecting both their physical barrier function and their intrinsic antimicrobial and antioxidant composition (Kahramanoğlu et al. 2019; Sarker and Grift 2021).

Enzymatic browning and lipid peroxidation are additional deterioration pathways coatings can help mitigate. Phenolic oxidation catalyzed by polyphenol oxidase is a primary cause of surface discoloration in fresh‐cut and mechanically damaged produce, and antioxidant‐enriched coatings have been shown to inhibit enzymatic browning by limiting oxygen availability and delivering reducing compounds to the tissue surface (A. K. Singh et al. 2022; W. Zhang et al. 2020). Limiting oxygen diffusion while delivering encapsulated antioxidants is a well‐documented, effective strategy for maintaining color and nutritional quality (Al‐Tayyar et al. 2020; Otoni et al. 2014). However, the stability of both the coating matrix and its bioactive constituents under high temperature is compromised. Increased thermal energy accelerates the degradation of phenolic compounds, essential oil components, and other labile actives, while simultaneously reducing the structural cohesion of the biopolymer network through accelerated chain mobility and increased permeability (M. Ali et al. 2025; Mondal et al. 2022). This dual degradation dynamic, in which thermal energy simultaneously degrades bioactive compounds and reduces biopolymer structural cohesion, defines the core design challenge for bioactive‐enriched warm‐chain coating systems. The challenge is not improving initial bioactive loading or encapsulation efficiency, though both matter. It is designing a delivery architecture in which the rate of bioactive release across the postharvest timeline is deliberately calibrated to match the commodity's actual microbial and oxidative pressure profile under warm‐chain conditions, so that protection is sustained precisely when and where deterioration pressure is highest. This is the quantitative design question that the controlled‐release systems pillar of the warm‐chain design framework addresses, and to the authors' knowledge, it has not been formally defined for any commodity‐coating combination in the 150 studies reviewed.

### 3.5 Implications for Performance Under Variable Environmental Conditions

The mechanisms governing edible coating functionality are highly interconnected and collectively sensitive to environmental variability. Gas exchange, moisture transfer, metabolic activity, and microbial dynamics do not operate independently. Changes in one domain propagate through the others in ways that can amplify overall deterioration. Under warm‐chain conditions, where temperature and humidity fluctuate rather than remain stable, these interactions become substantially more complex and less predictable (Maringgal et al. 2020; Ncama et al. 2018). Temperature‐induced increases in polymer chain mobility alter gas and water vapor diffusion coefficients simultaneously, while humidity‐driven swelling of the matrix changes mechanical properties that in turn affect the physical integrity of the coating layer (Arnon‐Rips and Poverenov 2018; Flores‐López et al. 2016). These material‐level changes occur against a background of shifting commodity physiology, as the same temperature and humidity fluctuations that degrade coating performance also accelerate respiration, ethylene production, and microbial growth within the coated commodity. The result is a compounding vulnerability in which coating performance declines precisely as the physiological demands placed on it increase.

This compounding vulnerability is not merely theoretical. Coatings that demonstrate strong performance under stable conditions may produce inconsistent and insufficient outcomes in real supply chains, with the degree of performance loss depending on both the specific material composition and the severity of environmental variability encountered (Garcia and Davidov‐Pardo 2021; Yousuf et al. 2018).

The analysis of coating behavior under warm‐chain conditions establishes a key design implication. Gas diffusivity, moisture transfer coefficients, respiration rates, and bioactive stability respond to environmental variables simultaneously in directions that amplify each other, so no coating property optimized under fixed laboratory conditions maintains function under warm‐chain variability. The performance loss in warm‐chain conditions is not random noise but the predictable output of materials encountering unplanned conditions. This indicates the need for a design framework where environmental dynamics are primary inputs rather than variability to control, and where performance specifications align with deployment conditions rather than measurement conditions.

## 4 Critical Limitations of Edible Coating Systems in Postharvest Applications

The systems‐design failure diagnosed earlier has four material‐level and operational components, examined in turn below. Each is necessary but insufficient alone, and together they establish that the performance gap is not a materials quality problem, formulation diversity problem, or one that incremental laboratory optimization can resolve.

### 4.1 Structural and Physicochemical Limitations of Biopolymer Matrices

Most edible coatings are derived from polysaccharides, proteins, and lipids, each with inherent structural weaknesses that limit their standalone functionality. Polysaccharide‐based coatings such as starch, cellulose derivatives, and natural gums generally exhibit good film‐forming ability but suffer from high water vapor permeability owing to the hydrophilic nature of their polymer networks, which allows water molecules to diffuse readily through the matrix and limits their effectiveness as moisture barriers (Table 1) (Galus et al. 2020; Panahirad et al. 2021; Yildirim‐Yalcin et al. 2022). This limitation is particularly consequential for high‐moisture commodities stored under warm and humid conditions, where the driving force for moisture loss is greatest, and the structural stability of the coating is most rapidly compromised.

The three‐axis trade‐off space—moisture barrier, gas exchange selectivity, and thermal‐hygroscopic stability—makes this pattern analytically explicit, and no biopolymer class excels on all three simultaneously. Polysaccharide‐based coatings, including starch, cellulose derivatives, and natural gums, score well on gas exchange selectivity through dense hydrogen‐bonded networks. Still, their hydrophilic nature drives water uptake and permeability increase under warm‐chain humidity, leaving them poor on moisture barrier and thermal‐hygroscopic stability (Table 1) (Galus et al. 2020; Kocira et al. 2021; Panahirad et al. 2021; Yildirim‐Yalcin et al. 2022). Protein‐based coatings offer moderate mechanical strength and gas barrier properties, but humidity‐induced structural change and thermal softening make them unreliable at upper warm‐chain temperatures, where barrier properties diverge from laboratory values (Nair et al. 2020; Yaashikaa et al. 2023). Lipid and wax components score well on moisture barrier through hydrophobic crystalline structure and thermal stability, but score poorly on gas exchange selectivity and impose anaerobic risk at warm‐chain respiration rates when applied at moisture‐protective thicknesses (Dhall 2013; Flores‐López et al. 2016; Sapper and Chiralt 2018; S. Singh et al. 2016). Chitosan offers antimicrobial activity and moderate gas regulation. Still, its moisture barrier declines under elevated humidity through polymer network plasticization, and its functional performance is further complicated by dependence on molecular weight and degree of deacetylation, introducing variability across studies and commodities (Kerch 2015; Nair et al. 2020; Romanazzi and Moumni 2022; Saberi Riseh et al. 2023). Gum arabic provides good emulsifying and film‐forming properties but lacks the mechanical strength and gas barrier performance for standalone warm‐chain application without supplementary cross‐linking or blending (Saha et al. 2017; Tahir et al. 2019; Tiamiyu et al. 2023); together with *Aloe vera* gel, it shows moderate performance across all three axes rather than strong performance on one, reflected in the warm‐chain validation outcomes in Table 2. No single‐material system, however, maintains gas exchange selectivity, moisture barrier integrity, and structural stability across the full warm‐chain temperature and humidity range (Table 2).

**TABLE 2:** Edible coating performance across warm‐chain and cold‐chain validation contexts.

Coating system category	Storage condition	Primary commodities validated	Key quality outcomes reported	Synthesized warm‐chain performance	Principal warm‐chain design limitation	References
Warm‐chain validated studies (storage temperature ≥ 18°C)						
Gum arabic single layer	Warm‐chain (20°C–25°C)	Tomato, mango, plum, guava (C)	Reduced weight loss, delayed respiration, suppressed microbial decay, preserved antioxidant capacity across multiple tropical commodity types	Consistent positive outcomes at 20°C–25°C across diverse climacteric commodities under ambient storage conditions	Insufficient mechanical strength as standalone coating above 25°C; moisture barrier requires composite development for reliability at upper warm‐chain temperatures	A. Ali et al. (2013); Daisy et al. (2020); Shakir et al. (2022); Saleem et al. (2020)
*Aloe vera* gel single layer	Warm‐chain (20°C–28°C)	Tomato, papaya, fig, mango (C)	Reduced weight loss and surface microbial load, delayed color change, extended shelf life; intrinsic antimicrobial activity supplements passive barrier function	Effective moisture and microbial control at 20°C–28°C; intrinsic bioactive compounds provide functional advantage where microbial pressure is highest under warm conditions	Compositional variability across gel sources introduces batch‐to‐batch inconsistency; phenolic bioactive components degrade above 30°C, reducing functional advantage at upper warm‐chain temperatures	Chauhan et al. (2015); Parven et al. (2020); Marpudi et al. (2011); Ortega‐Toro et al. (2017)
Gum arabic with antioxidant enrichment	Warm‐chain (20°C–23°C)	Plum, apricot (C)	Maintained antioxidant enzyme activity, reduced weight loss, delayed color deterioration, extended marketable shelf life	Effective at 20°C–23°C; antioxidant enrichment augments gum arabic baseline performance and extends functional protection duration under warm conditions	Antioxidant stability under warm conditions declines over storage duration; enrichment concentration requires commodity‐specific optimization	Shakir et al. (2022); S. Ali et al. (2021); Anjum et al. (2020)
Chitosan‐beeswax composite	Warm‐chain (20°C)	Strawberry (NC)	Reduced weight loss, maintained firmness and color, suppressed microbial growth; lipid component improved moisture barrier relative to chitosan alone	Effective at 20°C; lipid component provides moisture barrier advantage not achievable with chitosan alone under warm conditions	Anaerobic risk at warm‐chain temperatures requires precise lipid fraction calibration; efficacy at temperatures above 22°C not well‐characterized	Velickova et al. (2013)
*Aloe vera*–shellac composite	Warm‐chain (28 ± 2°C)	Tomato (C)	Maintained firmness, reduced respiration, delayed color development; shellac component substantially improved moisture barrier at elevated ambient temperatures	Effective at 28 ± 2°C; among the few systems with documented positive performance at the upper warm‐chain temperature range	Gas exchange compromise under high temperature requires precise shellac fraction calibration; limited commodity range validated	Chauhan et al. (2015)
*Aloe vera*–gum arabic composite with plant extract	Warm‐chain (25 ± 3°C)	Guava (C)	Reduced weight loss and browning, maintained antioxidant capacity and ascorbic acid, extended shelf life; multicomponent system addresses moisture, microbial, and oxidative deterioration simultaneously	Effective at 25 ± 3°C; multicomponent formulation shows additive functional benefits beyond either polymer alone	No single blend optimized all quality attributes simultaneously; plant extract selection requires commodity‐specific guidance	Anjum et al. (2020)
Guar–*Aloe vera* and guar–*Spirulina* composites	Warm‐chain (25 ± 2°C)	Mango (C)	Reduced respiration and weight loss; additive‐specific performance differences; *Aloe vera* blend superior for ascorbic acid; *Spirulina* blend superior for firmness and antioxidant retention	Effective at 25 ± 2°C; demonstrates importance of bioactive selection for target quality attribute under warm conditions	Different additives optimize different quality attributes; requires commodity‐ and attribute‐specific formulation decisions	Ebrahimi and Rastegar (2020)
Gum arabic–chitosan composite with ZnO nanoparticles	Warm‐chain (ambient)	Banana (C)	Enhanced antimicrobial efficacy, maintained firmness, delayed ripening; sustained cell membrane‐disruption antimicrobial activity	Effective under ambient conditions; nanoparticle reinforcement provides sustained antimicrobial advantage over non‐reinforced composites	Food safety and dietary exposure data under warm‐chain conditions entirely absent; no regulatory approval pathway established in target tropical markets	Le et al. (2021)
*Opuntia ficus‐indica* mucilage‐pectin composite	Warm‐chain (ambient)	Plum (C)	Reduced weight loss and respiration, inhibited cell wall softening enzymes, maintained antioxidant quality and color	Effective under ambient conditions; cell wall enzyme inhibition documented as a functional mechanism beyond simple barrier action	Thermal stability and humidity response profile entirely uncharacterized; warm‐chain behavior above 25°C unknown	Shinga and Fawole (2023)
Starch‐based single‐layer	Warm‐chain (20°C)	Banana, plum (C)	Delayed ethylene biosynthesis, reduced respiration and weight loss, prolonged chlorophyll retention; effective only at lower warm‐chain range	Effective at lower warm‐chain range (20°C) only; performance declines progressively above 22°C due to increased polymer chain mobility and hygroscopic swelling	Marked hygroscopic swelling above 65% RH; performance at 25°C–28°C significantly inferior to 20°C; unsuitable as standalone warm‐chain coating without modification	Thakur et al. (2018, 2019); Abdillah and Charles (2021)
Mixed‐condition studies, direct warm‐chain vs. cold‐chain performance comparison						
Gum arabic–chitosan composite	Mixed (13 ± 1°C and 25°C)	Banana (C)	Weight loss, firmness, respiration, and color evaluated under both cold and warm storage conditions; most direct quantified warm‐to‐cold performance comparison in the screened literature	Efficacy retained at 25°C but measurably reduced relative to 13°C cold storage; warm‐chain performance gap is real but partial, composite retains meaningful protective function at ambient temperature	Composite ratio optimization specifically for warm‐chain thermal stability not yet undertaken; performance at 28°C–35°C not investigated	Maqbool et al. (2011)
*Aloe vera*–chitosan composite with nanoparticles	Mixed (18 ± 1°C)	Banana (C)	Reduced weight loss and respiration, maintained antioxidant quality, suppressed microbial load; nanoparticle component extended antimicrobial activity duration	Effective at 18 ± 1°C; nanoparticle enrichment extends antimicrobial protection relative to composite without nanoparticles	Food safety and migration data under warm‐chain conditions absent; regulatory status in sub‐Saharan African markets not established	Odetayo et al. (2022)
CMC with moringa plant extract	Mixed (5°C and 21 ± 1°C)	Avocado, citrus (C, NC)	Weight loss, firmness, respiration, and microbial control assessed across cold and ambient storage conditions; seasonal and handling variability evaluated	Performance gap substantial and consistent; shelf‐life extension reduced under ambient conditions; seasonal and handling variability compounds performance inconsistency under warm conditions	Performance gap between cold and warm storage is large; warm‐chain resilience requires further composite development and bioactive enrichment to reduce the cold‐to‐warm performance differential	Tesfay et al. (2017); Kubheka et al. (2020)
Starch‐rosemary EO‐nanoparticle composite	Mixed (variable)	Peach (C)	Weight loss, firmness, microbial decay, and antioxidant quality evaluated across temperature conditions including warm and cold storage	Cross‐temperature performance stability demonstrated; encapsulated EO component retained activity under warm conditions where free EO degrades rapidly; strongest encapsulation advantage evidence in mixed‐condition literature	Food safety and migration profiling under warm‐chain conditions absent; scale‐up feasibility of encapsulation process not established	Bashir et al. (2023)
Chitosan‐gum composite with essential oil	Mixed (25 ± 5°C)	Peach (C)	Weight loss, firmness, microbial control, and shelf life maintained across variable temperature range including warm conditions	Positive outcomes maintained across variable temperature range; EO enrichment contributes antimicrobial resilience under warm conditions	Performance under sustained high ambient humidity not evaluated; thermal variability simulation does not replicate full warm‐chain humidity stress	Goswami et al. (2024)
Cold‐chain only studies, warm‐chain validation absent						
*Aloe vera*–chitosan composite	Cold‐chain (5°C)	Blueberry (NC)	Reduced weight loss and microbial load, maintained antioxidant quality, extended shelf life; strong cold‐chain evidence base	Not evaluated under warm conditions; strong cold‐chain evidence cannot be assumed to transfer given chitosan's documented humidity sensitivity under warm conditions	Warm‐chain validation entirely absent; most cited composite in the non‐climacteric fruit literature yet has no ambient temperature evidence	J. M. Vieira et al. (2016)
*Aloe vera* with salicylic acid	Cold‐chain (4 ± 1°C)	Citrus, orange (NC)	Inhibited microbial load, reduced chilling injury, maintained firmness and shelf life; salicylic acid enhanced systemic defense response	Not evaluated under warm conditions; salicylic acid‐mediated stress resistance mechanism warrants warm‐chain investigation given theoretical relevance to thermal stress responses	Warm‐chain validation absent; mechanism of action is relevant to warm‐chain physiology but has not been tested under warm conditions	Rasouli et al. (2019)
Gum arabic with antioxidant enrichment	Cold‐chain (4 ± 1°C)	Strawberry, blueberry (NC)	Maintained firmness, color, and antioxidant capacity; reduced microbial load; comprehensive quality retention under refrigerated conditions	Not evaluated under warm conditions; gum arabic's demonstrated warm‐chain efficacy across warm‐chain studies above cold‐chain evidence cannot predict warm‐chain performance; the demonstrated efficacy of gum arabic under warm‐chain conditions in other coating configurations supports prioritizing warm‐chain validation for this specific enrichment system	Warm‐chain validation absent despite strong prior evidence for gum arabic efficacy under ambient conditions in other commodity types	Tahir et al. (2018); Yang et al. (2019)
*Aloe vera*–protein‐plant extract composite	Cold‐chain (4°C)	Fresh‐cut orange (FC)	Reduced weight loss and microbial load, extended shelf life; protein component improved film cohesion on cut surfaces	Not evaluated under warm conditions; protein matrix thermal sensitivity above 30°C makes warm‐chain application of this system architecture unlikely without replacing the protein component	Protein component is a structural liability at warm‐chain temperatures; reformulation removing or replacing gelatin is a prerequisite for warm‐chain viability	Radi et al. (2017)
*Ficus‐indica* mucilage	Cold‐chain (4°C)	Fresh‐cut kiwifruit (FC)	Maintained firmness and surface color, retarded senescence, extended shelf life; mucilage adhesion effective on cut produce surfaces	Not evaluated under warm conditions; most promising warm‐chain candidate for fresh‐cut applications based on warm‐chain evidence for intact plum	Warm‐chain validation entirely absent for fresh‐cut application; highest‐priority warm‐chain validation gap for indigenous materials in the fresh‐cut category	Allegra et al. (2017)

*Note*: Performance assessments in the synthesized warm‐chain performance column represent the authors' synthesis across the reviewed studies for each coating system; they are not derived from a single source. Rows are ordered by storage condition category, warm‐chain (≥ 18°C) first, mixed condition second, cold‐chain (≤ 10°C) last, to make the validation gap and performance pattern directly visible within a single analytical structure. Warm‐chain performance is a synthesized qualitative assessment drawn from reported outcomes relative to uncoated controls.

Abbreviations: C, climacteric; FC, fresh cut; NC, non‐climacteric.

Single‐material coatings are not merely suboptimal. The evidence reviewed suggests that simultaneous optimization within any polymer class is physically constrained, as improving moisture barrier in polysaccharides requires increasing hydrophobicity through lipid blending, which alters gas transport properties, while improving thermal stability requires increasing cross‐linking density or crystallinity, which changes permeability. Whether this constraint is thermodynamically irreducible or an engineering challenge addressable through novel polymer architectures remains to be determined, but the consistent pattern across material classes in Table 1 indicates that composite design is necessary for warm‐chain applications rather than optional (Panahirad et al. 2021).

This structural analysis directly motivates the commodity‐specific coating architecture pillar of the warm‐chain design framework. Because no single material can satisfy all three performance axes simultaneously, coating design must begin from the specific axis weightings required by the target commodity, determined by its cuticle composition, respiration rate, and ethylene class, and work backward to the best composite architecture for those weightings under warm‐chain conditions. A climacteric fruit with high respiration at 25°C requires a formulation prioritizing gas exchange selectivity while tolerating moderate moisture loss, pointing toward a polysaccharide‐dominant composite with calibrated lipid fraction. A thin‐skinned non‐climacteric fruit with low natural surface wax requires a formulation prioritizing moisture barrier over gas restriction, pointing toward a lipid‐enriched composite with antifungal bioactive supplementation. The three‐axis trade‐off space thus provides the analytical foundation for deriving commodity‐specific coating architectures (Figure 3; Table 2).

**FIGURE 3:**
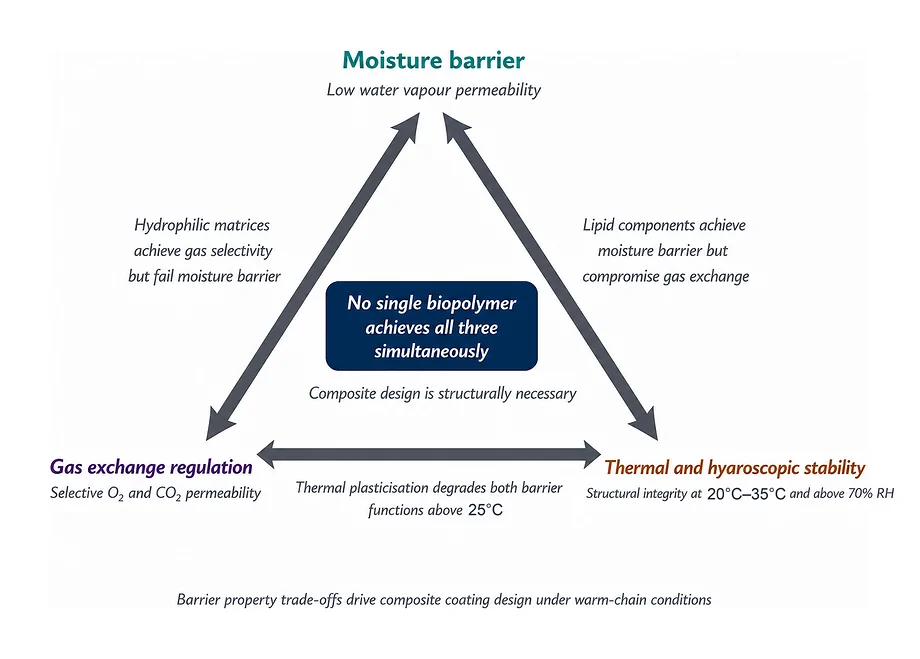
Barrier property trade‐offs among moisture resistance, gas exchange, and thermal‐hygroscopic stability that no single biopolymer class satisfies simultaneously under warm‐chain conditions, necessitating composite design.

### 4.2 Gas and Moisture Barrier Inconsistencies Under Variable Storage Conditions

Edible coating effectiveness depends critically on regulating gas exchange and moisture transfer simultaneously and consistently. Achieving this balance presents a persistent challenge, particularly under fluctuating environmental conditions that alter both coating properties and commodity physiology in parallel. Coatings designed to reduce respiration rates through restriction of oxygen diffusion risk inducing hypoxic conditions within the commodity if permeability is insufficient, leading to anaerobic respiration, ethanol accumulation, and the development of fermentative off‐flavors that render produce commercially unacceptable (Chavan et al. 2023; Pham et al. 2023). Coating‐induced atmosphere modification affects volatile compound development, with restricted gas exchange altering aroma profiles in strawberry, raising sensory acceptability concerns that compound the commercial risk of over‐restrictive formulations (Yan et al. 2018). Conversely, coatings with inherently high gas permeability may fail to suppress respiration sufficiently to delay ripening and senescence in high‐respiring commodities, particularly under warm temperatures where metabolic rates are elevated (Abdillah and Charles 2021; Galus et al. 2020).

Moisture regulation presents an equally complex challenge. Hydrophilic coatings absorb water under high relative humidity conditions, causing progressive swelling and plasticization of the polymer network that increases both water vapor and gas permeability over the storage period (Hasan et al. 2020; Maringgal et al. 2020). Under low relative humidity conditions, the same coatings may undergo rapid dehydration and physical contraction, promoting microcrack formation in the coating film. These microcracks create preferential pathways for direct moisture loss from the commodity surface (Armghan Khalid et al. 2022; Deng et al. 2017). Studies evaluating starch‐based coatings under ambient warm conditions have documented variable and often diminished effectiveness in controlling weight loss and maintaining firmness relative to refrigerated controls, reflecting strong dependence on environmental conditions that are difficult to stabilize in real supply chains (Abdillah and Charles 2021; Wigati et al. 2023). Maringgal et al. (2020) and Sharma et al. (2019) each document that coating performance on fresh‐cut produce is highly sensitive to storage temperature and humidity, with inconsistent outcomes reported across conditions in both studies. The gas and moisture barrier inconsistencies documented here indicate a design specification problem, not a materials quality issue. Characterizing coating permeability at a single temperature and humidity condition, then applying it across warm‐chain conditions, produces unreliable outcomes from incomplete specification. A coating whose gas permeability is characterized at 20°C and 60% relative humidity has only an assumed permeability at 30°C and 80% relative humidity. Closing this specification gap is a prerequisite for any warm‐chain coating validation protocol to produce transferable findings across studies and deployments.

### 4.3 Instability of Bioactive Compounds and Limitations in Controlled‐Release Functionality

The incorporation of bioactive compounds, including essential oils, plant polyphenols, organic acids, and antimicrobial peptides, is intended to extend the functional scope of edible coatings beyond passive barrier formation. However, maintaining their stability and bioavailability within the coating matrix throughout storage remains one of the field's most significant unresolved challenges. Bioactive compounds are sensitive to environmental conditions, with temperature, light exposure, and oxygen availability all capable of inducing degradation through oxidation, volatilization, and photolytic reactions that progressively reduce their concentration and efficacy during storage (Al‐Tayyar et al. 2020; Pandey et al. 2022). The rate of degradation is accelerated under warm‐chain conditions, precisely the environments where antimicrobial and antioxidant protection is most critically needed. The interaction between bioactive compounds and the polymer matrix introduces additional complexity. Matrix composition influences the molecular mobility of bioactive agents, their partitioning between the coating and commodity surface, and the kinetics of diffusion‐controlled release. Each of these factors alters the effective dose delivered to the commodity surface over time (Otoni et al. 2014; Yousuf et al. 2018). In many cases, matrix‐bioactive interactions result in rapid initial release followed by depletion of the active agent well before the end of the intended storage period, leaving the commodity unprotected during the later stages of supply chain transit when cumulative stress has already compromised surface integrity.

Nanoemulsion and encapsulation‐based strategies aim to improve bioactive retention and control release kinetics. These systems have demonstrated improved encapsulation efficiency and enhanced initial antimicrobial and antioxidant efficacy compared to conventional coating formulations. However, the sustained release of active compounds over extended storage periods under variable temperature and humidity conditions remains an unresolved technical challenge (de Oliveira Filho et al. 2021; Pirozzi et al. 2020). Similarly, antioxidant‐enriched coatings show strong initial performance in reducing enzymatic browning and oxidative quality loss, but their reproducibility and stability across different storage conditions and commodity matrices are often inconsistent (Mondal et al. 2022; W. Zhang et al. 2020). Compounding these difficulties, the concentration of bioactive compounds must be carefully optimized within narrow functional windows, as concentrations above sensory thresholds for taste, aroma, or texture can render produce commercially unacceptable despite achieving effective microbial or oxidative control (Romanazzi et al. 2017; Valencia‐Chamorro et al. 2011). The fundamental failure of current bioactive coating systems is not inadequate initial encapsulation efficiency. Rather, it is the absence of release kinetics designed to match physiological timelines of target commodities across supply chains under warm‐chain conditions. Existing systems are optimized for storage periods under which they were tested, rarely defined against actual supply chain transit times in specific market contexts. A coating whose bioactive depletes by Day 4 of a 10‐day transit has not failed through poor chemistry, but through absent calibration. Until release kinetics are designed prospectively against commodity‐specific protection timelines rather than characterized retrospectively from experiments, bioactive coating development will continue generating findings that cannot translate to reliable field performance.

### 4.4 Commodity‐Specific Variability and Limited Generalizability

A major structural limitation of edible coating research is the tendency to treat coating performance as primarily a materials science problem, when substantial evidence indicates that performance is equally determined by the physiological and surface characteristics of the target commodity. Differences in surface wax composition, stomatal density, cuticle thickness, and intrinsic respiration rate across fruit and vegetable species produce markedly different coating adhesion, permeability, and functional outcomes even when identical formulations are applied under identical conditions (Chockchaisawasdee et al. 2016; Riva et al. 2020). These differences are not minor sources of variability but fundamental determinants of coating efficacy that cannot be addressed through universal formulation approaches.

The consequences of this variability are documented across commodity categories in the reviewed literature, with the following pattern holding across studies. Composite coatings showing strong performance in non‐climacteric fruits such as strawberries and blueberries, where moisture retention and microbial control are key, may produce insufficient effects in climacteric fruits such as plums and bananas, where metabolic activity and ethylene dynamics create different performance demands (Pinzon et al. 2020; Tavassoli‐Kafrani et al. 2022). Fresh‐cut products present a further distinct challenge, as tissue disruption through cutting, peeling, or slicing dramatically increases susceptibility to microbial contamination and oxidative browning, requiring coatings that simultaneously provide enhanced barrier, antimicrobial, and antioxidant functionality that exceeds the requirements of intact commodities (Oms‐Oliu et al. 2010; Sharma et al. 2019).

Even within a single commodity category, surface characteristic differences between cultivars, growing conditions, and maturity stages can significantly affect coating uniformity, adhesion, and functional performance (Garcia and Davidov‐Pardo 2021; Shinga and Fawole 2023). This intra‐commodity variability limits the transferability of formulation outcomes between studies and complicates the development of standardized coating protocols for commercial application. The absence of a commodity classification framework not only slows formulation development but makes research literature structurally noncumulative. Since each commodity‐coating combination is optimized independently without a shared taxonomy, negative results are unreportable, positive results are non‐transferable, and the field is unlikely to develop transferable design principles without a common classification framework. A research program that has produced effective chitosan composite coatings for mango has not produced knowledge applicable to papaya without repeating the same experimental process, despite physiological similarities that a classification framework would make explicit. This is how an active and productive research field remains at the empirical stage decades after its foundational questions were posed.

### 4.5 Performance Gaps Under Real‐World and Warm‐Chain Conditions

One of the most consequential limitations of current edible coating research is not technical but epistemological. The evidence base for coating efficacy is overwhelmingly derived from studies conducted under controlled laboratory or refrigerated storage conditions, characterized by stable temperature, constant relative humidity, and minimal handling disturbance. These conditions do not reflect the variability encountered in practical horticultural supply chains, and the generalizability of findings from controlled studies to real‐world application contexts is therefore fundamentally uncertain (Chavan et al. 2023; Yaashikaa et al. 2023). Studies that have specifically evaluated coatings under ambient or fluctuating storage conditions provide a sobering contrast to laboratory outcomes. Available comparisons of coating performance under warm ambient versus cold controlled storage reveal substantial reductions in effectiveness under ambient conditions, with weight loss, decay incidence, and quality decline all progressing more rapidly despite the presence of coatings (Ebrahimi and Rastegar 2020; Wigati et al. 2023). The magnitude of performance degradation varies with coating type and commodity, and the limited number of direct comparisons precludes precise quantification of the performance gap across the field as a whole. However, the available evidence consistently points in this direction. Maqbool et al. (2011) report that gum arabic–chitosan coated banana showed 24% lower weight loss and 31% higher firmness than uncoated fruit after 33 days of combined cold storage and simulated ambient marketing. Ebrahimi and Rastegar (2020) document 31%–38% greater weight loss in guar–*Aloe vera* coated mango under ambient conditions versus cold storage over 21 days. Wigati et al. (2023) report that starch–basil oil coatings maintained mandarin firmness for 8 days under ambient conditions versus 14 days refrigerated, a 43% reduction in shelf‐life extension. The same pattern holds outside citrus and tropical fruit. Velickova et al. (2013) report 15%–20% lower weight loss in beeswax‐chitosan composite and three‐layer coated strawberry relative to uncoated fruit at 20°C, and Chauhan et al. (2015) report that a shellac–*Aloe vera* composite doubled tomato shelf life from 6 to 12 days at ambient temperature. Three further studies extend this evidence base. Shakir et al. (2022) report that a gum arabic–carboxymethyl cellulose biocomposite reduced tomato weight loss to 9.24% versus 14.04% in uncoated fruit after 20 days at 20°C, a 34% relative reduction. Shinga and Fawole (2023) report that 2% *Opuntia ficus‐indica* mucilage reduced banana weight loss to 10.24% versus 12.4% in uncoated fruit after 8 days at retail conditions, extending marketable life to 12 days. Thakur et al. (2018) report that a starch coating reduced banana weight loss to 3.18% versus 5.23% in uncoated fruit at day 10, extending the analysis period from 10 to 14 days before the uncoated control lost marketability. Across banana, mango, mandarin, strawberry, and tomato, coated fruit consistently outperforms uncoated fruit under ambient or warm‐chain storage. However, the metric reported differs by study, spanning weight loss, firmness, and days of shelf life, which is itself a symptom of the absence of standardized outcome reporting diagnosed in Section 4.4. These differentials are consistent but represent the current limit of quantitative synthesis from a heterogeneous evidence base. Standardized warm‐chain validation protocols, as specified in Pillar 4 of the proposed framework, are needed for generating a comparable evidence base for reliable performance ranges.

Beyond temperature and humidity, variability in handling practices, transportation methods, and storage infrastructure further compounds the inconsistency of coating performance in real supply chains. Physical abrasion during transport can disrupt coating film integrity, exposure to ethylene from mixed commodity storage can override coating‐mediated ripening suppression, and intermittent refrigeration creates repeated thermal cycling that accelerates the plasticization and structural degradation of hydrophilic coating matrices (Kubheka et al. 2020; Tesfay et al. 2017). These supply chain realities are rarely incorporated into experimental designs, creating a persistent and widening gap between the conditions under which coatings are evaluated and the conditions under which they must ultimately perform. Addressing this gap requires not merely incremental improvements in formulation but a fundamental reorientation of experimental design toward standardized warm‐chain evaluation protocols that incorporate realistic environmental variability as a core design variable rather than a source of noise to be controlled (Bhan et al. 2022; Maringgal et al. 2020). The cumulative result is a field whose evidentiary base is structurally unable to predict warm‐chain performance, not because the materials are poorly characterized, but because the conditions under which they have been characterized are systematically unrepresentative of the conditions in which they must perform. Temperature‐driven increases in polymer chain mobility, humidity‐driven plasticization, and accelerated bioactive degradation act simultaneously on the coating system, producing a warm‐chain performance gap that conventional coating designs, optimized for stable, refrigerated conditions, are not formulated to bridge (Figure 4; Table 2). This performance degradation, evident across material classes in the shelf‐life, weight loss, and decay comparisons above, is systemic rather than specific to any single biopolymer.

**FIGURE 4:**
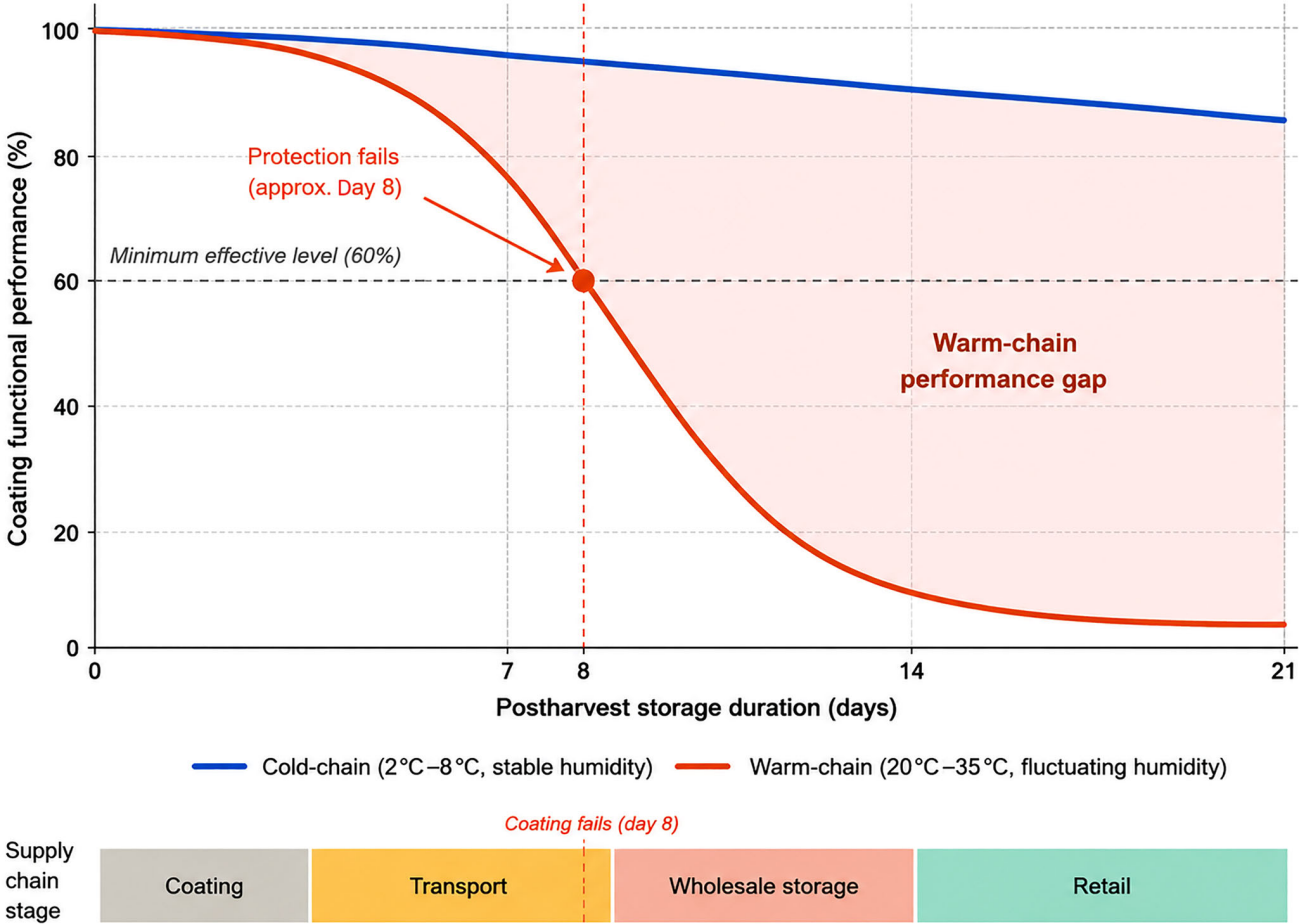
Schematic comparison of coating functional performance over 21 days of postharvest storage under cold‐chain versus warm‐chain conditions, illustrating the warm‐chain performance gap.

## 5 Advanced Formulation Strategies to Overcome Material and Performance Constraints

The evidence reviewed establishes that edible coatings' failure under warm‐chain conditions stems not from insufficient materials diversity, but from material systems designed without reference to thermal, hygroscopic, and physiological demands of warm‐chain deployment. The formulation strategies reviewed in this section address these structural constraints to varying degrees, as shown in Table 3. Still, their effectiveness depends on whether they were designed and validated against warm‐chain rather than controlled laboratory conditions. The synthesis evaluating each strategy against this criterion reveals a clear pattern in Table 3. Most of the six principal formulation strategies have absent or limited warm‐chain validation, indicating that recent innovation has improved performance more under controlled conditions than under actual required conditions (Figure 5).

**TABLE 3:** Formulation strategies for warm‐chain edible coatings, ranked by deployment readiness.

Formulation strategy	Representative systems	Functional gain over single‐polymer baseline	Warm‐chain performance evidence	Primary barrier to warm‐chain deployment	Deployment readiness	Critical research action
Indigenous composite systems (gum arabic and chitosan‐based)	Gum arabic–chitosan composite; gum arabic–CMC biocomposite; *Aloe vera*–gum arabic with plant extract; gum arabic–starch with essential oil	Synergistic improvement in mechanical strength, antimicrobial activity, and moisture regulation; multicomponent systems address gas, moisture, and microbial challenges simultaneously	Consistent positive outcomes across tropical climacteric commodities including mango, banana, guava, and tomato at 20°C–25°C; direct mixed‐condition comparison confirms efficacy retained at 25°C versus cold storage with quantified but acceptable performance reduction	Formulation ratio sensitivity under warm conditions not yet predictively characterized; performance at upper warm‐chain range (28°C–35°C) largely untested	High	Response‐surface optimization of composite ratios under warm ambient conditions; systematic validation at 28°C–35°C to establish upper performance boundary; Maqbool et al. (2010, 2011); Daisy et al. (2020); Shakir et al. (2022)
Lipid‐enriched composite systems	Chitosan‐beeswax; shellac–*Aloe vera*; alginate‐carnauba wax emulsion; chitosan‐alginate with olive oil	Superior moisture barrier relative to all‐polysaccharide alternatives; hydrophobic component confers structural stability under humid conditions that polysaccharides cannot achieve alone	All available warm‐chain validation confirms consistent moisture loss reduction and surface protection advantages; thermal stability of wax crystalline phase inherently well‐suited to warm‐chain temperature range	Anaerobic risk from gas exchange restriction is acutely elevated at warm‐chain temperatures where commodity respiration rates are highest; precise lipid fraction calibration is nontrivial	High (moisture barrier) Moderate (gas exchange safety)	Systematic O_2_ and CO_2_ permeability quantification at 20°C, 25°C, and 30°C for each lipid fraction proportion; commodity‐specific safe lipid loading limits linked to warm‐chain respiration rate data; Velickova et al. (2013); Chauhan et al. (2015); T. M. Vieira et al. (2021)
Bioactive‐enriched systems (plant extract and essential oil)	Gum arabic–thyme EO; chitosan‐lemongrass EO; *Aloe vera*–ascorbic acid; starch‐basil EO; *Aloe vera*–citrus peel extract; chitosan‐de‐oiled plant extract	Active antimicrobial and antioxidant protection beyond passive barrier function; multi‐mechanism spoilage suppression addresses both microbial and oxidative deterioration simultaneously	Positive warm‐chain antimicrobial and antioxidant outcomes confirmed across multiple commodity–bioactive combinations; cold‐chain studies continue to dominate the evidence base; performance at temperatures above 25°C remains poorly characterized	Rapid volatilization and oxidative degradation of EO components under warm temperatures reduces sustained efficacy; release kinetics not calibrated to warm‐chain postharvest timelines; sensory impact requires commodity‐specific threshold mapping	Moderate	Encapsulation‐based stabilization of EO components with warm‐chain release kinetics profiling; sensory threshold mapping at warm‐chain temperatures across priority tropical commodities; Ebrahimi and Rastegar (2020); Mondal et al. (2022); Goswami et al. (2024)
Controlled‐release encapsulation systems (microencapsulation and nanoemulsion)	Pectin‐cyclodextrin‐cinnamaldehyde; alginate‐nanoemulsion‐oregano EO; starch‐rosemary EO nanocapsules; chitosan nanoemulsion	Substantially improved bioactive retention and controlled release relative to free incorporation; encapsulation protection extends functional activity; initial antimicrobial efficacy superior to nonencapsulated equivalents	Very limited warm‐chain validation; available studies confirm short‐term antimicrobial efficacy under ambient conditions; sustained release under warm‐chain temperature and humidity variability remains uncharacterized across all systems	Sustained release kinetics under warm‐chain variability uncharacterized; designs optimized for cold‐chain rather than warm‐chain timelines; scale‐up of encapsulation processes to commercial throughput largely unexplored	Moderate (short‐term) Low (long‐term)	Warm‐chain release kinetics models linking temperature and humidity to release rate; redesign of encapsulation systems around warm‐chain commodity storage timelines; Otoni et al. (2014); de Oliveira Filho et al. (2021); Pirozzi et al. (2020); Bashir et al. (2023)
Multilayer and layer‐by‐layer systems	Chitosan‐pectin‐cyclodextrin multilayer; chitosan‐alginate emulsion bilayer; sequential polysaccharide‐lipid deposition; ternary starch‐chitosan–*Aloe vera* system	Structural capacity to independently optimize each functional layer; theoretical ability to decouple gas barrier, moisture barrier, and bioactive delivery within a single coating architecture; improved adhesion on hydrophilic cut‐fruit surfaces	Warm‐chain evidence is very limited; available evidence on hydrophilic commodity surfaces shows promise where conventional single‐layer coatings fail due to poor adhesion; layer stability under thermal cycling and physical handling stress not characterized	Layer deposition under warm‐chain conditions substantially more technically demanding than under laboratory conditions; interlayer delamination risk under thermal cycling largely uncharacterized; application method complexity limits commercial feasibility	Low	Warm‐chain layer stability characterization under thermal cycling and physical abrasion; development of simplified two‐layer systems with commercially viable application methods; adhesion profiling on tropical commodity surfaces at warm‐chain temperatures; Arnon‐Rips and Poverenov (2018); T. M. Vieira et al. (2021)
Nanoparticle‐reinforced matrices (metal oxide and bio‐nanocomposites)	ZnO–chitosan–gum arabic; TiO_2_‐alginate; ZnO‐xanthan hybrid; bio‐nanocomposite films; ZnO nanoparticle‐polysaccharide systems	Demonstrated enhancement of antimicrobial barrier properties and mechanical performance beyond what composite biopolymer systems achieve; superior structural rigidity and reduced gas permeability	Functional warm‐chain performance well‐supported across multiple tropical commodity studies; antimicrobial and quality‐retention outcomes confirmed under ambient conditions; structural barrier enhancement documented at warm‐chain temperatures	Critical deployment barrier is regulatory and food safety rather than functional: migration data, dietary exposure assessment, and ecotoxicological profiles under warm‐chain conditions absent across the literature; no regulatory approval pathway exists for nanoparticle‐reinforced edible coatings in major tropical producing regions	Low (deployment readiness) High (functional performance)	Systematic food‐contact migration studies under warm‐chain storage conditions; dietary exposure modeling for target consumer populations; regulatory pre‐submission dossier development for priority markets in sub‐Saharan Africa, South Asia, and Latin America; La et al. (2021); Nxumalo et al. (2022); Le et al. (2021); Odetayo et al. (2022)

*Note*: Deployment readiness ratings are the authors' synthesized assessments based on the reviewed evidence; they are not externally validated ratings. Rows are ordered from highest to lowest warm‐chain deployment readiness, defined as: High = positive warm‐chain validation across ≥ 2 tropical commodity types, food‐grade regulatory status confirmed in at least one target region, no unresolvable scale‐up barriers identified; Moderate = positive warm‐chain evidence for ≥ 1 commodity, food‐safety status established, ≥ 1 deployment barrier requiring defined resolution; Low = warm‐chain validation absent, or ≥ 1 deployment barrier identified as unresolvable within current regulatory or infrastructure constraints of target regions. References are embedded within the critical research action column for brevity.

Abbreviation: EO, essential oil.

^†^Nanoparticle‐reinforced systems carry split ratings reflecting the disjunction between demonstrated functional performance and regulatory deployment readiness; both dimensions are stated separately.

**FIGURE 5:**
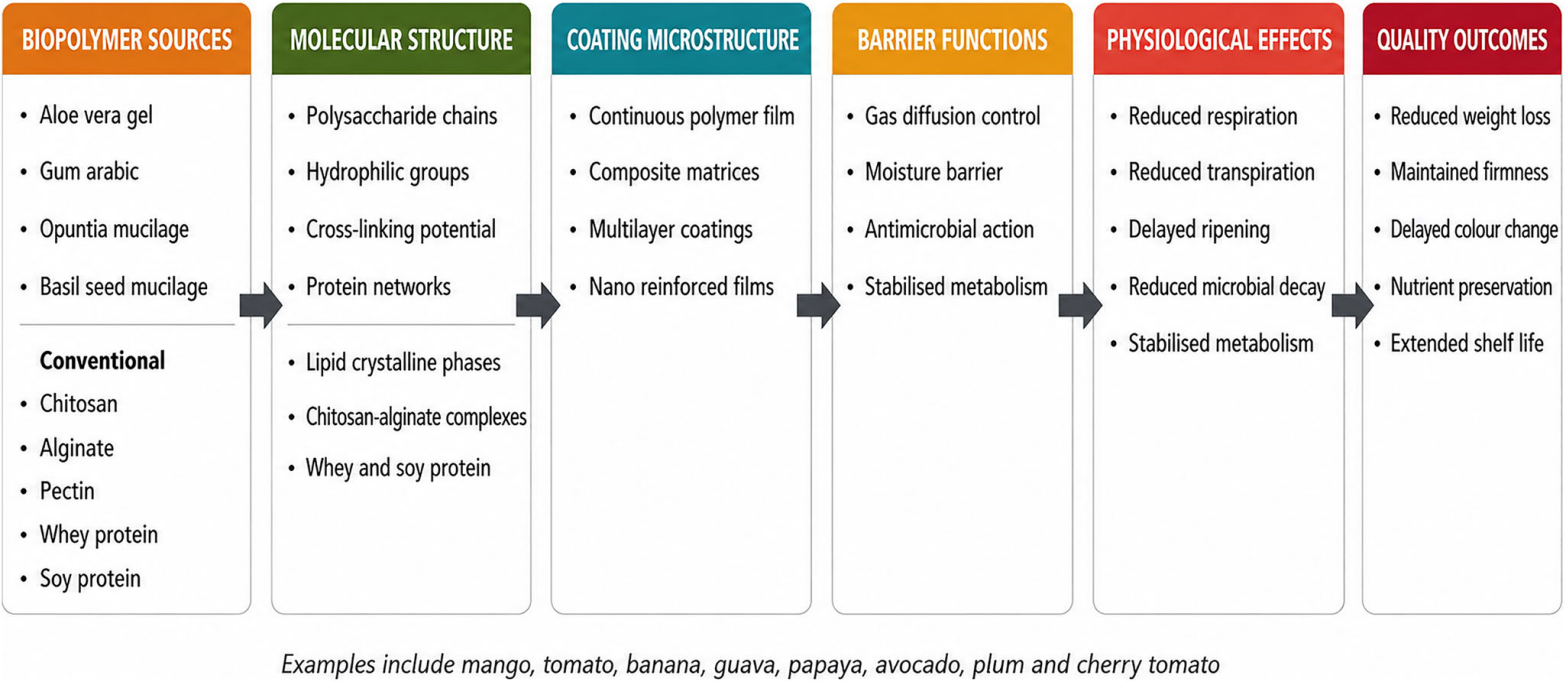
Structure–function–performance pathway in edible biopolymer coatings, from biopolymer source through molecular structure and microstructure to barrier function and quality outcome, illustrating the design rationale for composite systems.

### 5.1 Composite and Multilayer Systems to Resolve Single‐Material Limitations

The inability of single biopolymers to simultaneously provide effective moisture resistance, gas regulation, and mechanical stability has been the primary driver behind the development of composite and multilayer coating systems. Single‐material systems face fundamental physical trade‐offs in which improvements in one performance dimension, such as reduced water vapor permeability, are typically achieved at the cost of another, such as gas exchange selectivity. Current evidence indicates that simultaneous optimization of all required functional properties within a single polymer matrix is a fundamental trade‐off rather than merely a technical difficulty (Galus et al. 2020; Panahirad et al. 2021). Combining materials with complementary physicochemical properties lets composite formulations distribute functional requirements across multiple polymer components, overcoming the performance imbalances inherent in individual biopolymer systems.

Empirical evidence confirms composite systems' functional advantages across multiple commodity types. Coatings based on gum arabic and chitosan have demonstrated enhanced mechanical strength, improved surface adhesion, and strong antimicrobial activity in bananas, resulting in markedly reduced postharvest decay and superior quality retention relative to single‐component alternatives (Maqbool et al. 2011; Wani et al. 2021). Similarly, *Aloe vera* and chitosan composite systems have been shown to reduce respiration rates, suppress microbial growth, and maintain firmness in fruits including blueberries and mangoes, reflecting improved simultaneous control of gas exchange and moisture loss compared to either material applied alone (Shah and Hashmi 2020; J. M. Vieira et al. 2016).

More complex multicomponent formulations integrating structural biopolymers with plant‐derived bioactive extracts extend these benefits further. A composite coating combining gum arabic and *Aloe vera* gel with plant extracts significantly improved shelf life and antioxidant stability in guava stored under ambient conditions, demonstrating that multicomponent systems can integrate structural and functional benefits in ways that are unachievable with simpler formulations (Anjum et al. 2020). Similarly, ternary systems combining starch, chitosan, and *Aloe vera* have been reported to improve barrier properties and delay senescence in strawberries, with the *Aloe vera* component contributing both structural cohesion and intrinsic antimicrobial functionality (Ortega‐Toro et al. 2017; Pinzon et al. 2020). Incorporating lipid or shellac components within composite matrices substantially improves moisture barrier performance. Shellac‐enriched coatings reduced water loss and enhanced surface protection in tomato, addressing the poor moisture barrier property that represents the main weakness of hydrophilic polysaccharide matrices (Chauhan et al. 2015; T. M. Vieira et al. 2021).

Despite these advances, composite systems introduce their own challenges. Performance outcomes are highly sensitive to the ratios of component materials, the method and sequence of their combination, and the environmental conditions under which the coating is applied and subsequently stored (Chen et al. 2023; Dave et al. 2017; Garcia and Davidov‐Pardo 2021). These sensitivities show that composite systems mitigate single‐polymer limitations without eliminating the challenge of designing formulations that maintain functional balance across variable storage environments. The warm‐chain validation evidence for composite systems in Table 3 reveals a consistent pattern. Composites with the strongest warm‐chain performance use materials tolerant to high temperature and humidity, particularly gum arabic and *Aloe vera* gel composites tested in tropical conditions, and guar gum–chitosan composites evaluated under mixed conditions (Goswami et al. 2024). Systems with the weakest validation use hydrophilic polysaccharide matrices without lipid or cross‐linking components, though optimized starch‐based coatings delay banana ripening under ambient conditions (Thakur et al. 2018, 2019). This confirms that composite design alone cannot ensure warm‐chain performance. The materials combined and their structural rationale determine whether the system achieves warm‐chain resilience or merely redistributes hygroscopic vulnerabilities across polymer components.

The evidence on composite system warm‐chain performance contains a significant contradiction. Studies on gum arabic–chitosan composites at 20°C–25°C show positive outcomes, with Maqbool et al. (2011) noting maintained fruit quality at 25°C compared to refrigerated storage. However, starch‐based coatings fail above 22°C due to humidity‐driven plasticization (Thakur et al. 2018, 2019). These findings may indicate material‐class differences, with chitosan's cationic network more humidity‐resilient than starch's matrix, or differences in respiration rates, coating thickness, or humidity variability. The lack of standardized warm‐chain validation protocols, as noted in Pillar 4, prevents determining the correct explanation. This is the type of impasse the framework aims to resolve.

### 5.2 Bioactive‐Enriched Coatings to Improve Functional Stability and Efficacy

Postharvest quality instability under microbial and oxidative stress has prompted research into enriching edible coating matrices with bioactive compounds for active, sustained protection beyond passive barrier function. The incorporation of essential oils, plant polyphenols, organic acids, and antimicrobial peptides has shown potential in reducing microbial decay and oxidative deterioration across commodities (Pandey et al. 2022; Yousuf et al. 2021). Bioactive‐enriched systems offer functional breadth, since one formulation can address microbial spoilage, enzymatic browning, and oxidative quality loss through complementary mechanisms. Gum arabic and chitosan composite coatings have demonstrated significant suppression of fungal pathogens in banana under warm ambient storage conditions, with the combined antimicrobial action of these materials providing protection where fungal pressure is highest (Le et al. 2021; Maqbool et al. 2011). Chitosan‐based active coatings applied to pineapple have demonstrated effective antimicrobial protection and shelf‐life extension under ambient warm conditions, confirming the broad commodity applicability of chitosan's antimicrobial function beyond climacteric tropical fruits (Basumatary et al. 2021). *Aloe vera* coatings combined with ascorbic acid have been shown to substantially reduce microbial load while maintaining physicochemical quality attributes in strawberries and date fruits, with the synergy between *Aloe vera*'s intrinsic antimicrobial compounds and ascorbic acid's antioxidant activity producing outcomes superior to either agent applied independently (Alkaabi et al. 2022; Sogvar et al. 2016).

Stress‐modulating signaling compounds represent an additional and underexplored category of bioactive addition. The inclusion of salicylic acid within *Aloe vera* coating matrices has been reported to activate systemic defense responses within climacteric tissues, enhancing resistance to oxidative stress and delaying senescence in citrus fruits through upregulation of antioxidant enzyme activity and suppression of ethylene biosynthesis (Nicolau‐Lapeña et al. 2021; Rasouli et al. 2019). This mechanism is relevant to warm‐chain applications as salicylic acid‐mediated defense activation operates through temperature‐independent pathways that reduce physiological deterioration under ambient thermal stress, not only cold storage conditions. Similarly, antioxidant formulations with tea polyphenols and black and green tea extracts inhibit polyphenol oxidase activity and reduce enzymatic browning in fresh‐cut oranges and minimally processed products, extending color stability throughout storage (Odetayo et al. 2022; Radi et al. 2017).

Nanostructured approaches offer further potential for improving bioactive delivery and retention. Coatings incorporating zinc oxide nanoparticles within chitosan and gum arabic composite matrices have demonstrated enhanced antimicrobial efficacy and delayed ripening in banana, with the nanoparticle component providing sustained antimicrobial activity through direct membrane disruption and reactive oxygen species generation at microbial cell surfaces (La et al. 2021; Nxumalo et al. 2022). Nanoemulsion‐based delivery systems represent a complementary strategy, with the capacity to encapsulate hydrophobic bioactive compounds, including essential oils, within colloidal droplets that improve their dispersion, stability, and controlled release within the coating matrix (de Oliveira Filho et al. 2021; Khan et al. 2023; Zambrano‐Zaragoza et al. 2018) (Figure 6). Zinc oxide‐reinforced xanthan gum hybrid systems have demonstrated comparable antimicrobial reinforcement effects to chitosan‐based nanocomposites, confirming that the nanoparticle enhancement mechanism is not confined to a single biopolymer matrix class (Joshy et al. 2020). Starch‐based nanoparticle systems with quercetin have shown bioactive retention and quality preservation in grapes, extending encapsulation evidence to polyphenolic compounds (Zeng et al. 2024). The main unresolved issue in bioactive coating science is bridging initial antimicrobial and antioxidant efficacy with sustained protection during the warm‐chain postharvest period. Studies show encapsulation improves initial retention versus free incorporation, but none demonstrates sustained bioactive activity matched to commodity‐specific protection timelines under warm‐chain conditions. This gap requires shifting from characterizing system release under test conditions to specifying required release timing and rates to maintain protection against microbial and oxidative pressures for defined commodities in specific supply chains. This design philosophy shift has not occurred for any commodity‐coating combination in the literature.

**FIGURE 6:**
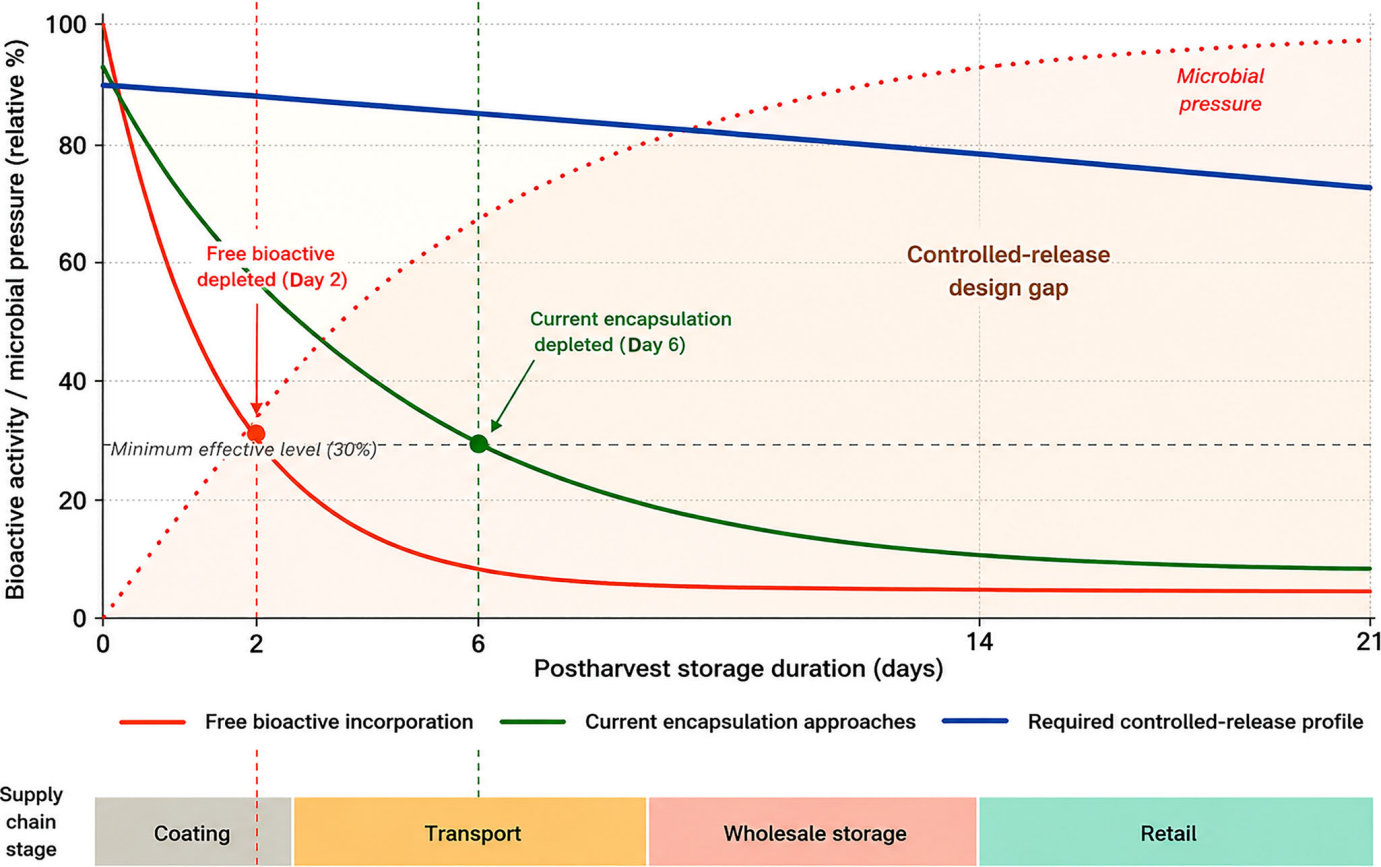
Schematic bioactive release profiles over 21 days of storage for free bioactive incorporation, current encapsulation approaches, and the release profile required for sustained protection, illustrating the controlled‐release design gap.

### 5.3 Commodity‐Specific Optimization to Address Variability in Coating Performance

The evidence reviewed in Sections 3 and 4—that coating effectiveness is shaped by the physiological and surface characteristics of target commodities—has driven a shift toward crop‐specific formulation strategies, replacing the assumption that formulations optimized for one commodity category will perform adequately across others. Differences in surface morphology, cuticular wax composition, stomatal density, and metabolic behavior that distinguish commodity categories impose distinct performance requirements on coating systems, necessitating tailored approaches for each commodity context (M. Singh et al. 2024; Tavassoli‐Kafrani et al. 2022). In climacteric fruits including tomato, mango, and banana, coating systems are primarily designed to regulate respiration rates and delay ethylene‐mediated ripening. Gum arabic‐based coatings have been particularly well‐documented in this context, demonstrating consistent capacity to maintain firmness, delay color development, and preserve antioxidant capacity in tomato stored under both ambient and controlled conditions, reflecting strong compatibility between gum arabic film properties and the physiological requirements of climacteric ripening management (A. Ali et al. 2010, 2013; Nawab et al. 2017). Composite coatings applied to mango and guava have similarly demonstrated improved control of ripening processes and sustained quality retention, with the combination of structural and bioactive components addressing both the gas exchange requirements and the microbial vulnerability characteristic of these tropical climacteric fruits (Anjum et al. 2020; Saleem et al. 2020).

Non‐climacteric fruits and berries have different coating requirements, as the absence of a ripening climacteric means postharvest quality management focuses on suppressing moisture loss, limiting microbial colonization, and preserving surface integrity rather than modulating ripening dynamics. *Aloe vera*‐based coating systems have shown strong performance across this category, producing consistent reductions in weight loss and decay in strawberries, blueberries, and carambola, reflecting suitability for high‐moisture, thin‐skinned commodities where surface protection against transpiration and microbial ingress is the primary requirement (Gol et al. 2015; Sogvar et al. 2016; Tahir et al. 2018; J. M. Vieira et al. 2016). Fresh‐cut products represent a distinct commodity category, characterized by tissue damage‐induced susceptibility to microbial contamination, enzymatic browning, and moisture loss that places demanding performance requirements on coating systems. Formulations applied to fresh‐cut kiwifruit and citrus segments demonstrate the importance of integrating antioxidant and antimicrobial functionality within a coating matrix to address multiple deterioration pathways activated by tissue disruption (Allegra et al. 2016; S. Kumar et al. 2021; Oms‐Oliu et al. 2010). These three commodity categories' distinct requirements confirm that commodity‐specific optimization is a prerequisite for reliable coating efficacy, not a refinement of universal approaches, and that the absence of classification frameworks linking commodity physiology to coating design is a fundamental structural gap in the field (Chockchaisawasdee et al. 2016; Riva et al. 2020). The empirical approach currently used to achieve this, in which each commodity‐coating combination is independently tested until an effective formulation is found, is a research design that cannot scale to the number of commodity categories, cultivars, and supply chain contexts for which warm‐chain coating solutions are needed. A classification framework based on measurable physiological and surface parameters is therefore not an organizational convenience but the structural requirement for making coating design knowledge cumulative rather than perpetually restarting.

### 5.4 Indigenous and Dryland‐Adapted Biomaterials as Context‐Relevant Innovation Sources

The search for coating materials that combine functional performance with contextual relevance for warm‐chain supply chains in low‐income production regions has increasingly focused on indigenous and dryland‐adapted biomaterials. These materials offer properties distinct from conventional synthetic or imported biopolymers through local availability, cultural familiarity, and adaptation to high‐temperature, variable‐humidity environments. This adaptation profile better matches the thermal and humidity cycling of warm‐chain supply chains than the stable conditions under which most coating materials are optimized (Tahir et al. 2019; Tiamiyu et al. 2023).

Gum arabic, derived from *Acacia* species native to the semiarid Sahel region of Africa, has emerged as the most extensively warm‐chain validated indigenous coating biopolymer, with consistent positive outcomes documented across multiple tropical commodity types under ambient storage conditions (Table 2). Table 2 confirms that gum arabic consistently appears in the warm‐chain validated section, providing stronger evidence for warm‐chain deployment than most composite systems despite its simple single‐layer formulation. Its well‐documented emulsifying capacity, strong film‐forming ability, and high solubility at practical processing concentrations make it technically well‐suited to coating applications. At the same time, its widespread availability across sub‐Saharan Africa and its long‐standing global use as a food additive are favorable starting conditions for regulatory acceptance, though jurisdiction‐specific approval status across warm‐chain producing regions has not been systematically verified (Section 6.5.2) (Mahfoudhi and Hamdi 2015; Maqbool et al. 2011). Its effectiveness spans many commodities such as tomato, banana, guava, mango, avocado, and persimmon, consistently delaying ripening, reducing respiration, and improving antioxidant retention. Integrating it with chitosan, starch, or plant extracts further enhances mechanical properties and versatility (A. Ali et al. 2010; El‐Gioushy et al. 2022; Kawhena et al. 2021; Maqbool et al. 2010). *Aloe vera* gel is a complementary biomaterial with a unique functional profile, offering structural and bioactive contributions to coating performance. Composite coatings of *Aloe vera* gel with lemon peel extract and calcium chloride delayed ripening and maintained quality in banana and mango under ambient conditions, highlighting *Aloe vera*'s versatility for multicomponent bioactive integration (Hajebi Seyed et al. 2021; Jodhani and Nataraj 2021). Its gel matrix contains polysaccharides, including acemannan that contribute to film formation and moisture regulation. At this time, its diverse suite of phenolic compounds, anthraquinones, and organic acids provides intrinsic antimicrobial and antioxidant activity that persists within the coating matrix throughout the storage period (Kahramanoğlu et al. 2019; Sarker and Grift 2021). Applications across strawberries, blueberries, citrus, papaya, guava, fresh fig, peach, and plum have demonstrated consistent reductions in microbial load, delayed senescence, and maintained physicochemical quality, with the multifunctional nature of the *Aloe vera* matrix allowing a single coating component to address simultaneous postharvest challenges (Guillén et al. 2013; Marpudi et al. 2011; Nicolau‐Lapeña et al. 2021; Rasouli et al. 2019; Rehman et al. 2020; Sogvar et al. 2016).

Beyond gum arabic and *Aloe vera*, *Opuntia* cactus mucilage has attracted growing research interest as an alternative matrix material with strong film‐forming ability, compatible barrier properties, and particular suitability for fresh‐cut and stone fruit applications in semiarid producing regions where *Opuntia* species are abundantly available (Allegra et al. 2016, 2017; Shinga and Fawole 2023; C. Rodrigues et al. 2023). The use of such regionally sourced materials directly addresses the need for low‐cost, locally available, and environmentally adapted coating solutions in regions where access to advanced storage infrastructure is limited and import‐dependent coating materials represent a prohibitive cost barrier to adoption (Bill et al. 2014; James and Zikankuba 2017; Kubheka et al. 2020). Gum arabic and *Aloe vera* gel have more credible warm‐chain validation than most composite systems. However, they remain among the least characterized materials in coating literature regarding their physicochemical behavior under warm‐chain conditions. Their film‐forming capacity, water vapor permeability, gas barrier performance, and thermal stability across 20°C–35°C and 55%–85% relative humidity have not been fully quantified, so the physicochemical characterization needed to understand the basis of that advantage is lacking. Closing this characterization gap is the key near‐term research priority for warm‐chain coating science in sub‐Saharan Africa, as the materials are locally available. The required measurements—film permeability, mechanical strength, and thermal‐hygroscopic behavior under standard conditions—use established characterization methods (Table 1) rather than specialized equipment unavailable in the region, so that results would provide biopolymer‐specific parameters needed for predictive modeling of relevant commodity‐material combinations.

### 5.5 Design Priorities for Coatings Under Variable and Warm‐Chain Conditions

The limitations in conventional coating systems and innovations discussed previously converge on a central design imperative. Effective edible coating systems must be designed for real‐world storage environments from the start, rather than optimized under controlled laboratory conditions and adapted later for practical use. As established in Section 4.5, coating functionality under ambient and fluctuating warm‐chain conditions is consistently reduced relative to refrigerated storage controls, so warm‐chain design requires a different approach rather than adjustments to laboratory‐derived formulations (Ebrahimi and Rastegar 2020; Wigati et al. 2023). Technical priorities must be specified as quantitative design requirements. Gas permeability must be specified across 20°C–35°C using Arrhenius‐derived diffusion coefficients for the biopolymer‐commodity system, ensuring O_2_ and CO_2_ exchange remains within aerobic respiration windows across the warm‐chain temperature range. Structural integrity must be maintained above the plasticized *T*_g_ of the coating matrix under warm‐chain humidity, with *T*_g_ characterization at 55%–85% RH. Bioactive release must be designed against microbial growth and oxidative pressure timelines under warm‐chain conditions, specifying release rate and duration to maintain protection above effective thresholds throughout the supply chain (Bhan et al. 2022; J. M. Vieira et al. 2016). The reviewed literature does not yet provide these specifications for any commodity‐coating combination, confirming that this is the design gap the proposed four‐pillar framework is structured to address.

Studies under variable storage conditions reveal which coating characteristics are most durable under warm‐chain stress. Formulations with hydrophobic lipid components or cross‐linked polysaccharide networks show greater structural stability under thermal and hygroscopic variation than hydrophilic single‐polymer systems, while coatings with encapsulated rather than free bioactive compounds demonstrate improved retention of antimicrobial and antioxidant activity at elevated temperatures (Moalemiyan and Ramaswamy 2012; Nandane et al. 2017). The most promising coating systems are not those achieving optimal performance under fixed laboratory conditions, but those demonstrating capacity to maintain function across commodities, environments, and supply chain scenarios that characterize real postharvest systems. Achieving this balance requires that warm‐chain performance be established as a primary design criterion rather than a secondary validation endpoint (A. Ali et al. 2013). Table 3 reveals that indigenous composite systems using gum arabic and *Aloe vera* demonstrate the strongest validation records due to testing in tropical conditions. Controlled‐release encapsulation and multilayer systems show weaker validation, having been evaluated mainly in cold‐chain laboratory settings. This inverse relationship between technological sophistication and warm‐chain validation is the field's most consequential structural signal. It indicates that the direction of innovation and the direction of deployment needs are misaligned, and that resolving this misalignment requires not more advanced materials but a design framework that establishes warm‐chain conditions as primary inputs rather than secondary validation contexts.

## 6 Strategic Research Gaps and a Four‐Pillar Design Framework for Warm‐Chain Edible Coatings

The four‐pillar framework proposed here is organized around a reversal of the design logic that has governed edible coating development. The pillars form a proposed design framework based on reviewed evidence, not an established protocol. Its effectiveness depends on coordinated implementation of all four pillars together, which has not yet been demonstrated for any single commodity‐coating system, and the measurable indicators specified for each pillar in Table 4 are targets for future validation rather than criteria the framework has already met. Each pillar identifies a research direction that would make warm‐chain coating design predictive and cumulative instead of empirical and iterative. Rather than beginning from what materials can do and evaluating whether their performance transfers to warm‐chain conditions, the framework begins from what warm‐chain environments require and derives the formulation, release, classification, and validation specifications that those requirements impose. Each pillar addresses one category of requirement that the evidence reviewed has shown current coating design does not systematically meet. The pillars are ordered by their dependency relationships: the commodity‐specific architecture pillar provides the physiological parameters that the predictive formulation modeling and controlled‐release pillars require as design inputs, while the warm‐chain validation pillar provides the performance standard against which all three upstream pillars are tested. This sequencing means the framework is not a parallel research agenda but a structured design pipeline in which advances in one pillar enable and constrain advances in the others (Figure 7).

**TABLE 4:** Four‐pillar warm‐chain design framework, evidence base, and proposed outcome indicators for future validation.

Research gap	Pillar	Specific design requirement	Key variables	Measurable outcome indicators	References
Empirical, trial‐and‐error formulation dominates coating development	Predictive formulation modeling	Define permeability targets based on commodity respiration and storage conditions	O_2_ permeability, CO_2_ permeability, WVP, temperature (20°C–35°C), RH (55%–85%), respiration rate	Reduction in formulation iterations; ≥ 20%–30% improvement in first‐pass coating performance under ambient storage; maintenance of aerobic respiration (no ethanol accumulation)	Dhall (2013); Jafarzadeh et al. (2021); Kocira et al. (2021); Maringgal et al. (2020)
Mismatch between coating permeability and commodity physiology	Predictive formulation modeling	Match gas diffusion to respiration kinetics of climacteric and non‐climacteric fruits	Respiration rate, internal O_2_/CO_2_ levels, coating thickness, diffusivity coefficients	Stable internal gas composition; delayed climacteric peak; reduced respiration rate without anaerobic shift	Wilson et al. (2019); Caleb et al. (2013); Sapper and Chiralt (2018)
Rapid depletion or instability of bioactive compounds under warm conditions	Controlled‐release systems	Design release profiles aligned with storage duration and environmental conditions	Release rate constant, degradation rate, matrix–bioactive interaction, temperature sensitivity	Sustained antimicrobial activity ≥ 70%–80% over storage duration; consistent antioxidant capacity; no early depletion	Al‐Tayyar et al. (2020); Otoni et al. (2014); Valencia‐Chamorro et al. (2011)
Uncontrolled release leading to sensory defects or ineffective protection	Controlled‐release systems	Optimize encapsulation and diffusion pathways within coating matrix	Encapsulation efficiency, diffusion coefficient, concentration threshold, sensory limits	Effective microbial inhibition without sensory deviation; controlled release over defined time window	Galus et al. (2020); Nair et al. (2020)
High variability in coating performance across commodities	Commodity‐specific coating architecture	Classify commodities based on physiological and structural traits	Respiration class, cuticle composition, surface morphology, moisture loss rate	Reduced variability in coating performance across commodity groups; ≥ 25% improvement in reproducibility	Pinzon et al. (2020); Chockchaisawasdee et al. (2016); Riva et al. (2020)
Poor coating adhesion and inconsistent surface coverage	Commodity‐specific coating architecture	Adapt formulation to surface properties and wax composition	Surface roughness, wettability, coating viscosity, polymer compatibility	Uniform coating distribution; improved adhesion; reduced patchiness and permeability variation	Tahir et al. (2019); Ncama et al. (2018)
Lack of validation under realistic supply chain conditions	Warm‐chain validation protocols	Develop standardized testing under dynamic environmental conditions	Temperature fluctuation, RH variation, storage duration, handling stress	Consistent performance under simulated supply chain; ≤ 15% performance drop compared to controlled conditions	Maringgal et al. (2020); Thakur et al. (2018); Yousuf et al. (2018)
Over‐reliance on cold‐chain evaluation methods	Warm‐chain validation protocols	Incorporate ambient storage and thermal cycling into evaluation frameworks	Temperature cycles (20°C–35°C), RH cycles, time intervals	Demonstrated coating stability under cyclic conditions; validated shelf‐life extension under ambient storage	Ncama et al. (2018); FAO (2023)
Lack of integration across formulation, functionality, and validation	Framework integration	Combine all four pillars into a unified design pipeline	Cross‐variable interaction: permeability, release, commodity traits, environment	Coating systems that maintain ≥ 80% functional performance across full supply chain duration	Otoni et al. (2017); Al‐Tayyar et al. (2020); Jafarzadeh et al. (2021)

*Note*: This table represents a proposed design framework; the four pillars and their specifications are the authors' conceptual contributions grounded in, but not directly derived from, any single reviewed study. Each row represents one pillar of the proposed framework, moving from what the evidence shows is missing to what must be done and how progress would be measured. Outcome indicators are proposed research targets derived from performance thresholds in the highest‐performing studies reviewed, not regulatory standards, and no coating‐commodity combination has yet met them experimentally.

**FIGURE 7:**
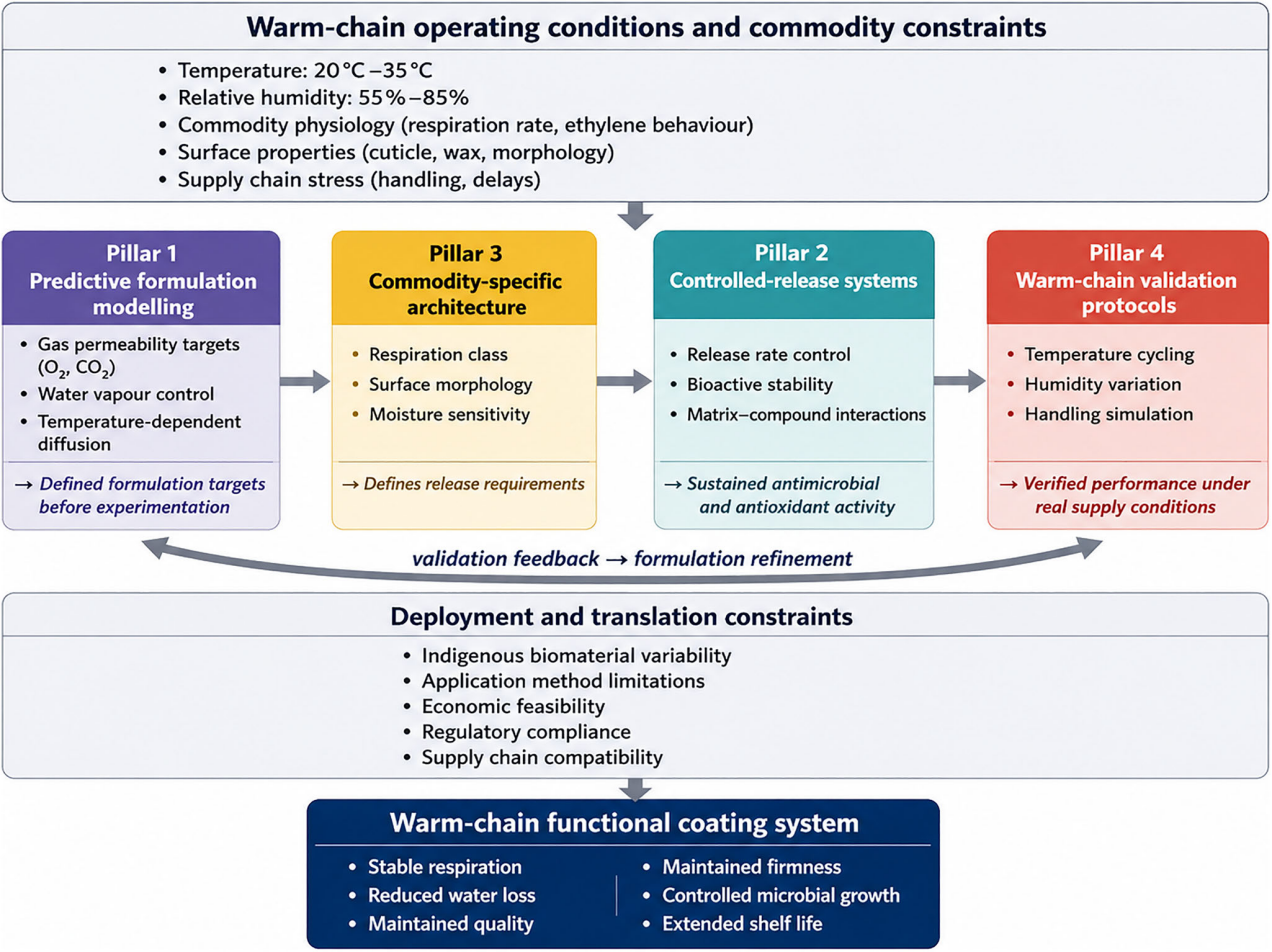
Four‐pillar warm‐chain design framework—predictive formulation modeling, commodity‐specific coating architecture, controlled‐release calibration, and standardized warm‐chain validation—structured as a dependent design pipeline rather than parallel research directions.

### 6.1 Predictive Formulation Modeling

The central limitation of current edible coating development is its reliance on empirical optimization. Composite and nano‐enabled systems have improved functional performance. However, formulation strategies remain largely dependent on iterative trial‐and‐error approaches, so improvements achieved within a specific material system or commodity context cannot be systematically transferred or reproduced across different storage environments (Table 3; de Souza et al. 2019; Galus et al. 2020). A predictive formulation approach requires explicitly linking coating transport properties to commodity physiology under defined environmental conditions. Gas movement across the coating must be aligned with the respiration behavior of the product, maintaining sufficient oxygen availability while preventing excessive carbon dioxide accumulation. Water vapor movement must, at the same time, be restricted enough to reduce mass loss without inducing condensation or structural instability under high relative humidity. Temperature strongly influences both processes, as increases within the 20°C–35°C range alter diffusion rates and polymer mobility, changing gas permeability and water vapor transmission together (Sapper and Chiralt 2018; Y. Zhang et al. 2024).

Diffusion modeling is used extensively in synthetic packaging systems, but equivalent approaches have not been systematically applied to biopolymer coatings, which limits the field's ability to define formulation targets in advance rather than discover them empirically. Multilayer and nanocomposite systems offer additional degrees of control over gas and moisture transport. Their performance, however, depends on interfacial interactions, phase distribution, and diffusion pathways that are rarely quantified during formulation design (Saberi Riseh et al. 2023; Zambrano‐Zaragoza et al. 2018). Data‐driven approaches such as response surface methodology and machine learning‐assisted optimization have demonstrated measurable improvements in formulation efficiency for multivariable problems, particularly where interactions between polymer composition, environmental conditions, and commodity behavior are nonlinear (Nandane et al. 2017). These approaches, though, are not yet integrated into standard coating development workflows and are typically applied after the fact rather than as predictive design tools. The key research priority is to determine whether diffusion‐based models incorporating temperature‐dependent transport properties, glass transition behavior, and water activity can predict water vapor permeability within an acceptable error margin across 20°C–35°C conditions for representative biopolymer systems. Gas permeability predictions must, in parallel, be validated against the respiration‐driven oxygen and carbon dioxide dynamics of target commodities. Demonstrating this predictive capability for a single, well‐characterized biopolymer class would provide the foundation for extending model‐based formulation across coating systems, reducing empirical dependency and enabling transferable design strategies.

### 6.2 Controlled‐Release Systems Calibrated to Postharvest Timelines

The effectiveness of bioactive‐enriched edible coatings depends not only on the presence of antimicrobial and antioxidant compounds, but on the rate and duration of their release. Under warm‐chain conditions, elevated temperature accelerates both degradation and diffusion of bioactive compounds, often resulting in rapid initial release followed by insufficient activity during later stages of storage (Al‐Tayyar et al. 2020; Otoni et al. 2014). This temporal mismatch limits the ability of coatings to provide sustained protection across typical supply chain durations. Release behavior is governed by interactions between the bioactive compound and the polymer matrix, as well as by environmental conditions such as temperature and relative humidity. Strong matrix–compound interactions may delay release but reduce immediate efficacy, while weak interactions can lead to rapid depletion of active compounds (Radi et al. 2017; Zambrano‐Zaragoza et al. 2018). In addition, increases in temperature within the 20°C–35°C range enhance molecular mobility and diffusion rates, further accelerating release and shortening the functional lifespan of the coating (de Oliveira Filho et al. 2021; J. P. Rodrigues et al. 2021).

Current approaches, including nanoemulsion and microencapsulation systems, have improved initial retention of bioactive compounds. However, their ability to maintain controlled release under fluctuating warm‐chain conditions remains limited (Chaudhary et al. 2020; La et al. 2021). Effective design therefore requires aligning release kinetics with the duration of storage and handling, ensuring that bioactive concentrations remain above effective thresholds throughout the supply chain without exceeding sensory limits (Mondal et al. 2022; Valencia‐Chamorro et al. 2011). The key research priority is to determine whether the release rate of encapsulated bioactive compounds follows predictable temperature‐dependent behavior across 20°C–35°C and whether these release profiles can be matched to microbial growth dynamics of target commodities. Demonstrating this alignment would establish the basis for coating systems capable of sustained antimicrobial and antioxidant protection under warm‐chain conditions.

### 6.3 Commodity‐Specific Coating Architectures

A major limitation in edible coating development is the assumption that formulations can be transferred across commodities with minimal adjustment. In practice, coating performance depends on a commodity's surface structure, cuticle composition, respiration behavior, and moisture dynamics, each of which shapes adhesion, gas exchange, and water loss (Chockchaisawasdee et al. 2016; Pinzon et al. 2020; Riva et al. 2020). For example, climacteric fruits with high respiration rates require coatings that maintain sufficient oxygen permeability to prevent anaerobic metabolism, while commodities prone to rapid moisture loss require stronger water vapor barriers to limit dehydration (Hasan et al. 2020). In addition, variations in surface wax composition and roughness add a further, independent source of variation, since they affect coating uniformity and permeability regardless of respiration behavior, so two commodities with similar respiration profiles can still respond differently to the same formulation (A. Ali et al. 2013; Ncama et al. 2018). Fresh‐cut products add a distinct category of demand rather than a further degree of the same problem. Tissue disruption increases oxygen exposure, accelerates water loss, and raises susceptibility to microbial contamination, so these products need coatings that combine barrier function with antimicrobial protection and oxidation control, a combination intact commodities do not require (M. Singh et al. 2024; Yousuf et al. 2021).

A commodity‐specific design approach requires grouping produce based on shared physiological and structural characteristics, including respiration class, surface properties, and sensitivity to moisture loss. These groupings can then be used to define formulation classes that are tailored to each category, reducing the need for repeated empirical optimization and improving the transferability of coating systems. The key research priority is to determine whether a limited set of measurable parameters, such as respiration rate, cuticle properties, and transpiration thresholds, can be used to classify major tropical commodities into coating design categories, and whether such classification improves the consistency and predictability of coating performance compared to conventional empirical approaches.

### 6.4 Standardized Warm‐Chain Validation Protocols

A major barrier to translating edible coating technologies into practical application is the reliance on evaluation methods that do not reflect real supply chain conditions. Most studies assess coating performance under stable temperature and humidity, often under refrigerated storage, which does not capture the variability encountered in warm‐chain environments (Ncama et al. 2018; Thakur et al. 2018). In practice, horticultural commodities in warm regions are exposed to temperature fluctuations within the range of 20°C–35°C, relative humidity variation, and repeated handling during harvesting, packing, transportation, and marketing. These conditions influence coating structure, alter gas and water vapor movement, and affect the stability of incorporated bioactive compounds, leading to performance outcomes that differ from those observed under controlled laboratory conditions (Maringgal et al. 2020; Wilson et al. 2019).

Evidence comparing ambient and controlled storage indicates that coating effectiveness often declines under non‐refrigerated conditions, with increased weight loss, faster softening, and higher microbial incidence reported across multiple commodities (Basumatary et al. 2022; Yousuf et al. 2018). Such comparisons remain rare, however, and the absence of a standardized testing framework prevents the extraction of generalizable design parameters from them. A standardized protocol should build the same conditions directly into the experimental design rather than treating them as an afterthought, cycling temperature and humidity across the full warm‐chain range and simulating mechanical handling events, while tracking weight loss, firmness, respiration rate, microbial load, and bioactive retention throughout the storage period.

Validation should also extend beyond single‐site laboratory experiments to multilocation trials across warm‐chain producing regions, where environmental variability and handling practices differ substantially (Hodges et al. 2011; Karoney et al. 2024). Real‐time monitoring of temperature, humidity, and coating integrity during storage and transport would further improve the accuracy of these assessments under actual supply chain conditions (Gidado et al. 2024; Hoffmann et al. 2025). Establishing standardized protocols along these lines would allow consistent comparison across studies and provide a reliable basis for coating design and optimization.

### 6.5 Interdependence of the Four Pillars and Constraints on Translation

The four pillars described above are interdependent components of a unified design system rather than independent research directions, and their effectiveness depends on coordinated implementation rather than the isolated optimization of each in turn. Predictive formulation modeling defines the target gas and water vapor transport properties required to maintain physiological stability of the commodity under defined environmental conditions (Hasan et al. 2020; Sapper and Chiralt 2018). These targets are constrained by commodity‐specific characteristics, including respiration rate, cuticle properties, and surface morphology, which determine how coatings interact with the product and influence gas exchange and moisture loss (Chockchaisawasdee et al. 2016; Riva et al. 2020). Controlled‐release systems extend this design by aligning bioactive delivery with storage duration and microbial or oxidative dynamics (Al‐Tayyar et al. 2020; Otoni et al. 2014). Standardized validation protocols then determine whether the combined system performs under fluctuating temperature, humidity, and handling conditions representative of warm‐chain supply environments (Maringgal et al. 2020; Ncama et al. 2018).

Modifications made to reduce water loss alter gas diffusion and affects respiration, while modifying matrix structure to control bioactive release influences adhesion and permeability (Jafarzadeh et al. 2021; Kocira et al. 2021). Validation under dynamic conditions provides a mechanism for identifying these interactions and guiding iterative refinement of coating design. The effectiveness of the four‐pillar pipeline depends on two enabling conditions spanning all pillars. The first is the physicochemical characterization of indigenous biomaterials, which determines whether regionally available materials can be incorporated into the predictive modeling and controlled‐release design needed for Pillars 1 and 2. The second is the engineering, economic, and regulatory feasibility of translating coating systems into operational warm‐chain supply contexts, which determines whether Pillar 4 validation outcomes can be implemented at commercial scale. Table 4 consolidates the four pillars against their evidence base, associated research gaps, and measurable outcome indicators.

#### 6.5.1 Role of Indigenous Biomaterials in Warm‐Chain Coating Design

Coating design for warm‐chain systems depends not only on functional performance but also on material availability, cost, and regional relevance. Indigenous biomaterials such as gum arabic and *Aloe vera* gel are widely accessible in many warm‐chain producing regions, but their integration into predictive coating design remains limited by a shared characterization gap. Most studies report empirical improvements in shelf life or microbial stability without measuring the water vapor permeability, gas diffusion behavior, thermal stability, and rheological properties that Pillar 1 modeling and controlled‐release design require (Mahfoudhi and Hamdi 2015; Tiamiyu et al. 2023).

Gum arabic's emulsifying capacity and demonstrated compatibility with bioactive compounds make it a strong candidate for composite coatings under warm ambient conditions, though its interactions with other biopolymers under temperature and humidity variation have not been systematically evaluated (Kawhena et al. 2021; Wani et al. 2021). *Aloe vera* gel offers antimicrobial and film‐forming properties, but its composition varies with species, cultivation conditions, and processing method, which reduces reproducibility across studies and compounds the same characterization gap (Nicolau‐Lapeña et al. 2021; Sarker and Grift 2021). Closing this gap for both materials, and extending the same characterization effort to other regionally available biomaterials, is what would move indigenous coatings from empirical application toward systematic, predictive design.

#### 6.5.2 Scalability and Translation Across Supply Chains

The translation of edible coating technologies from laboratory studies to commercial application depends on engineering, economic, and regulatory factors that are largely absent from current research designs. Application method is a primary constraint, and not one unique to edible coatings: Bassey et al. (2021) show that scaling from laboratory to commercial application is a consistent bottleneck across postharvest technology categories generally. For coatings specifically, the mechanism is uniformity: coverage and thickness achieved through laboratory‐scale dipping or spraying are difficult to reproduce at high‐throughput packing house speeds, and the resulting variability in coverage can materially affect gas exchange and moisture loss (Hasan et al. 2020; Prasad et al. 2022). This directly undermines systems designed within the predictive formulation modeling pillar and can defeat controlled‐release strategies calibrated to an assumed uniform application. Failure to plan for this at the design stage produces coatings that are technically effective in the laboratory and operationally impractical on a packing line (Affognon et al. 2015; Karoney et al. 2024).

Economic feasibility is rarely evidenced. Studies rarely report cost–benefit analyses, leaving producers without the data needed to assess the economic case for adoption (Kitinoja et al. 2011; Parfitt et al. 2010), and this gap is total for the two most field‐relevant systems in this review. No published source reports material cost per kilogram of produce treated for gum arabic or *Aloe vera*‐based coatings in sub‐Saharan Africa, South Asia, or Latin America. As an illustrative reasoning exercise rather than a data‐derived estimate, applying typical raw‐material prices to the application rates in Table 2 suggests costs on the order of $0.02 to $0.15 per kilogram, a range that would be affordable for smallholder operations if postharvest loss reduction of 15%–25% is achieved. However, this figure requires direct verification before use in any feasibility assessment. For gum arabic specifically, this order of magnitude is broadly consistent with reported Sudanese farmgate and aggregation prices of $0.70 to $2.00 per kilogram of raw material before dilution to coating‐strength concentrations (Dayoub 2025). However, comparable sourced pricing for *Aloe vera* gel and for the finished, diluted coating formulation was not identified in the reviewed literature. The harder gap sits one level up: no study has modeled the break‐even loss reduction required to justify adoption for any specific commodity‐market system, a calculation that needs commodity price, loss rate, and coating cost reported together, which they currently are not.

Regulatory frameworks vary across regions, and compliance requires consideration during formulation development rather than post hoc evaluation (Romanazzi et al. 2017; W. Zhang et al. 2020). Hence, alignment with existing supply chain infrastructure is essential. Coating systems must be compatible with current handling, storage, and transportation practices, including packing house operations and distribution logistics. Failure to account for these constraints results in technologies that are technically effective but operationally impractical (Affognon et al. 2015; Karoney et al. 2024). Regulatory status varies across target regions. Chitosan, gum arabic, and *Aloe vera* gel are approved food additives or GRAS‐listed ingredients in major jurisdictions, including the United States and European Union, and are traditionally consumed or used as food ingredients across sub‐Saharan Africa, South Asia, and Latin America, which plausibly presents a lower regulatory barrier than systems built on novel bioactives or nanoparticles. However, country‐specific food‐grade or novel‐food approval status for these materials has not been systematically verified in the 150 studies reviewed and would require jurisdiction‐by‐jurisdiction confirmation before informing a deployment decision. For nanoparticle‐reinforced coatings specifically, no formal regulatory pathway yet exists in these markets, and the absence of migration data and dietary exposure assessments is the main deployment constraint for that strategy class, as Table 3 reflects.

These constraints are not external to the framework. They must be designed into each pillar from the outset. A coating system that satisfies every pillar requirement but cannot be applied uniformly at packing house throughput, lacks a cost–benefit case, or has not cleared regional food safety compliance has not solved the warm‐chain deployment problem, only its laboratory equivalent. Closing that gap across sub‐Saharan Africa, South Asia, and Latin America will also require coordinated infrastructure between national agricultural research systems and universities, an institutional challenge to be planned alongside the research program itself.

### 6.6 A Six‐Phase Pipeline for Operationalizing the Four‐Pillar Framework

The four pillars described in Sections 6.1–6.4 specify what a warm‐chain coating system must achieve. They do not specify the order in which a research or development team proceeds from an untested commodity to a validated, deployable system. This section supplies that order as a six‐phase implementation pipeline (Figure 8), which Table 5 applies to six representative warm‐chain commodities.

**FIGURE 8:**
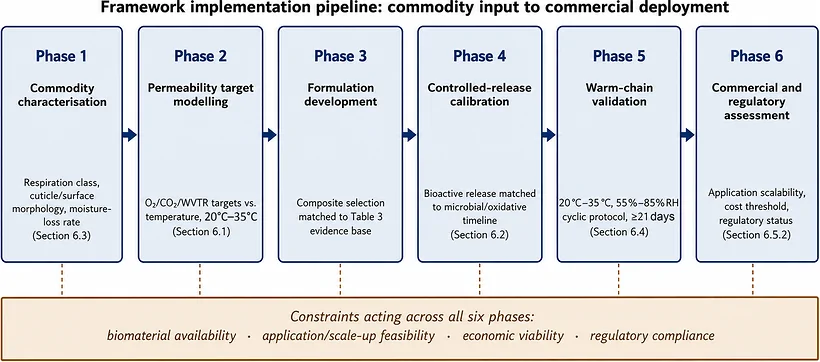
Six‐phase implementation pipeline operationalizing the four‐pillar framework in Figure 7, from commodity characterization through formulation, calibration, and validation to commercial and regulatory assessments.

**TABLE 5:** Commodity‐specific implementation matrix for representative warm‐chain horticultural commodities.

Commodity	Principal warm‐chain challenge	Recommended coating system (evidence base)	Warm‐chain validation condition	Primary commercial/scale‐up issue	Supporting evidence
Mango (climacteric)	High respiration rate and rapid weight loss during ambient transport	Guar–*Aloe vera* or guar–*Spirulina* composite (attribute‐specific selection); gum arabic single layer as baseline	25 ± 2°C, ambient RH (Table 2)	Additive‐specific performance requires an attribute‐specific formulation decision before uniform‐batch commercial application	Ebrahimi and Rastegar (2020); A. Ali et al. (2013)
Banana (climacteric, ethylene‐sensitive)	Ethylene‐driven ripening acceleration; chilling sensitivity precludes cold storage as a ripening brake	Gum arabic–chitosan composite, with or without ZnO nanoparticle reinforcement	Ambient warm‐chain temperature (Table 2)	Nanoparticle‐reinforced variants face an unestablished regulatory pathway and absent dietary exposure data in target markets (Section 6.5.2)	Le et al. (2021); Thakur et al. (2018, 2019)
Tomato (climacteric)	Respiration/ethylene regulation and moisture retention at the upper warm‐chain temperature range	*Aloe vera*–shellac composite, the system validated at the highest temperature of those reviewed	28 ± 2°C (Table 2)	Shellac fraction requires precise calibration at scale; validated commodity range remains narrow	Chauhan et al. (2015); A. Ali et al. (2013, 2010)
Guava (climacteric)	Browning, weight loss, and antioxidant degradation under ambient storage	*Aloe vera*–gum arabic composite with plant extract	25 ± 3°C (Table 2)	Plant extract selection requires commodity‐specific guidance; no single blend optimizes all quality attributes simultaneously	Anjum et al. (2020)
Papaya (climacteric)	Rapid softening and high microbial decay risk under humid conditions	*Aloe vera* single layer or *Aloe vera*‐based composite	20°C–28°C (Table 2)	Gel compositional variability between sources introduces batch‐to‐batch inconsistency at scale	Parven et al. (2020); Marpudi et al. (2011)
Fresh‐cut produce (wound physiology)	Enzymatic browning and elevated respiration from wounding; high microbial exposure at cut surfaces	Antioxidant‐ and antimicrobial‐enriched composite coatings	Standardized warm‐chain protocols specific to fresh‐cut produce not yet established (Section 6.4 gap)	Coating application method (dipping/spraying) compatibility with commercial fresh‐cut processing lines is largely unvalidated at throughput scale	Oms‐Oliu et al. (2008); J. P. Rodrigues et al. (2021)

*Note*: This table translates the four‐pillar framework (Table 4) and the Phases 1–6 implementation pipeline (Section 6.6; Figure 8) into commodity‐level recommendations for six representative warm‐chain commodities. Recommended coating systems and validation conditions are drawn directly from the warm‐chain‐validated evidence already synthesized in Table 2; this table adds no new primary data. Commercial/scale‐up issues restate constraints already identified in Sections 4.5, 6.5, and 6.5.2 for each commodity specifically. The matrix is a translation layer for practitioners and is not a substitute for commodity‐ and market‐specific feasibility assessment.

Phase 1 begins with commodity characterization. Before any formulation decision is made, the target commodity's respiration class, cuticle and surface morphology, and baseline moisture‐loss rate must be established, following the physiological parameters set out in Section 6.3. These parameters become the fixed inputs to every downstream phase. Phase 2 converts these inputs into permeability targets: using the temperature‐dependent diffusion relationship established in Section 3.1, oxygen, carbon dioxide, and water vapor transmission targets are specified as functions of temperature across the full 20°C–35°C warm‐chain range, rather than as single values measured at one reference temperature.

Phase 3 selects and develops the composite formulation expected to meet the Phase 2 targets, drawing on the functional gains and deployment readiness ratings already compiled in Table 3. Phase 4 calibrates the release kinetics of any incorporated bioactive compound against the commodity's actual microbial and oxidative pressure timeline, following the design logic set out in Section 6.2, so that protection is sustained when deterioration pressure is highest rather than concentrated in the first days of storage.

Phase 5 subjects the resulting system to standardized warm‐chain validation, cycling temperature and relative humidity across the 20°C–35°C and 55%–85% ranges for a minimum of 21 days, following the protocol gap identified in Section 6.4. Phase 6 assesses whether the validated system can be deployed commercially: application method compatibility with packing house throughput, the cost–benefit threshold discussed in Section 6.5.2, and confirmed regulatory status in the target market.

The six phases are not a one‐pass sequence. A system that fails Phase 5 returns to Phase 3 or Phase 4 with the failure mode treated as new design information; a system that fails Phase 6 on cost or regulatory grounds returns to Phase 3 with a narrower materials palette. This iterative structure is what allows the framework to accumulate transferable knowledge across commodities rather than restarting empirically for each new commodity‐coating combination, the obstacle to a cumulative research literature identified in Section 4.4. Applied to six commodities spanning the climacteric fruit types and the fresh‐cut category that dominate warm‐chain horticultural trade, Table 5 identifies, for each, the specific evidence gap that Phase 5 or Phase 6 has not yet closed.

These research priorities divide by time horizon rather than by pillar. The near‐term priorities, achievable within one to two years using established characterization methods rather than new instrumentation, are the physicochemical characterization of gum arabic and *Aloe vera* gel across the warm‐chain range (Section 5.4) and the commodity classification scheme linking respiration class and surface parameters to coating architecture (Section 6.3); both can proceed immediately from evidence already reviewed here. The longer‐term priorities, requiring sustained multi‐year investment because they depend on data accumulated across multiple commodity‐coating systems before a general model can be built and tested, are the predictive diffusion modeling linking temperature‐dependent permeability to commodity respiration (Section 6.1) and the standardized multisite warm‐chain validation protocol (Section 6.4). Controlled‐release calibration (Section 6.2) sits between the two horizons: matching release kinetics to a single commodity's protection timeline is achievable in the near term, but generalizing that calibration across commodity classes is a longer‐term undertaking that depends on the classification scheme above being in place first.

### 6.7 Limitations of the Evidence Base and the Proposed Framework

This review relies on narrative synthesis rather than meta‐analysis, because heterogeneity in outcome metrics, storage conditions, and commodity types across the 150 studies reviewed precluded meaningful quantitative pooling. Quantitative statements in this review, including the magnitude of the warm‐chain performance gap in Section 4.5, are therefore based on the available comparative studies rather than formal effect‐size estimates, and the review cannot assess effect‐size variability or publication bias across the wider literature using formal statistical methods.

A related risk is that the reviewed literature may itself be biased toward positive outcomes. Because coating research is not organized around a shared commodity classification framework, each coating‐commodity combination is evaluated independently, negative results are less likely to be published than positive ones, and the reviewed evidence base may consequently overestimate coating efficacy and underestimate the severity of the warm‐chain performance gap. The commodity‐specific coating architecture and standardized warm‐chain validation protocols proposed in Sections 6.3 and 6.4 would mitigate this risk by providing a structured reporting framework in which negative results contribute to cumulative knowledge rather than remaining unpublished.

The evidence underlying the four‐pillar framework is drawn primarily from tropical and subtropical horticultural commodities under the 20°C–35°C, 55%–85% RH warm‐chain conditions characteristic of sub‐Saharan Africa, South Asia, and Latin America. Its applicability to temperate‐zone commodities experiencing warm‐chain conditions during seasonal or retail distribution, or to cold‐chain commodities experiencing intermittent thermal cycling, has not been evaluated in the reviewed literature and would require separate validation before the framework is extended to those contexts.

More broadly, the four‐pillar framework and the six‐phase pipeline in Section 6.6 have been developed against the evidence synthesized in this review rather than against field‐scale trials conducted across the diversity of regions, commodities, and supply chain systems in which warm‐chain postharvest handling actually occurs. Local variation in ambient conditions, commodity cultivar and maturity, and supply chain infrastructure and handling practice can each shift the specific formulation and validation targets a given deployment requires. Field‐scale validation across representative regional and commodity–supply chain combinations remains necessary before the framework's recommendations are applied at commercial scale.

## 7 Conclusion

This review argues that edible coating underperformance in warm‐chain horticultural systems is a design problem, not a materials problem. Gas diffusivity, moisture transfer, respiration rate, and bioactive stability all respond to environmental variables in ways that amplify under warm‐chain conditions, so formulations optimized in the laboratory do not reliably transfer to the field. The reviewed literature indicates that no single biopolymer satisfies moisture barrier performance, gas exchange selectivity, and thermal‐hygroscopic stability simultaneously, making composite design an evidence‐based necessity rather than a preference. Bioactive release kinetics compound the problem when calibrated to storage duration rather than to a commodity's actual protection timeline, and the absence of a commodity classification framework keeps the resulting research noncumulative; each coating‐commodity combination is optimized independently, with findings that rarely transfer to the next. Composite formulations, nanostructured delivery systems, and indigenous biomaterials have each measurably outperformed single‐polymer systems under controlled conditions. None of that progress is sufficient for warm‐chain deployment without a framework that integrates these gains around deployment conditions rather than laboratory ones.

Adopting this framework changes what counts as progress in edible coating research. Under the current laboratory‐centered paradigm, progress is performance improvement under controlled conditions. Under this framework, progress is demonstrated functional integrity under warm‐chain variability, release kinetics matched to commodity‐specific protection timelines, classification systems that make formulation knowledge cumulative across commodity categories, and validation protocols that produce transferable findings rather than site‐specific results. These are not higher standards applied to the same research design. They are a different research design, organized around deployment conditions rather than measurement conditions. Without this reorientation, the field will keep producing findings that are rigorous in their experimental context and irrelevant to the warm‐chain supply chains where postharvest loss is greatest, and smallholder farmers and low‐income consumers will keep bearing that loss, not for want of effective materials, but for want of a framework to deploy them reliably. This review proposes that framework.

This review makes three contributions, distinguished from the five prior reviews compared in the Introduction by its focus on warm‐chain deployment rather than materials cataloging. First, it synthesizes evidence from polymer physics, release kinetics, and commodity physiology to diagnose warm‐chain coating failure as a systems‐design problem rather than a material deficit, formalizing this diagnosis into the three‐axis trade‐off set out above. Second, it proposes the four‐pillar warm‐chain design framework, with specified dependencies, measurable indicators, and a prioritized research agenda, as the basis for a cumulative research program in place of independent empirical studies. Third, it operationalizes this framework into a six‐phase implementation pipeline and a commodity‐specific application matrix, providing a structured pathway from formulation to field deployment that future work can test directly (Section 6.6; Table 5; Figure 8). The warm‐chain environments of sub‐Saharan Africa, South Asia, and Latin America are simultaneously the greatest need and the most demanding test of coating functionality. There, the shift from material‐driven innovation to integrated, deployment‐oriented design is not a long‐term ambition but the immediate condition for coating science to deliver postharvest outcomes at the scale smallholder farmers and low‐income consumers require.

## Author Contributions

**Olaoluwa Omoniyi Olarewaju**: conceptualization, investigation, writing – original draft, writing – review and editing, visualization, validation, methodology, formal analysis, data curation, project administration. **Olufunke Omowumi Fajinmi**: investigation, writing – original draft, writing – review and editing.

## Funding

The authors have nothing to report.

## Conflicts of Interest

The authors declare no conflicts of interest.

## Data Availability

Data sharing does not apply to this article because no new data were created or analyzed in this study.
